# Phage-delivered sensitisation with subsequent antibiotic treatment reveals sustained effect against antimicrobial resistant bacteria

**DOI:** 10.7150/thno.42573

**Published:** 2020-05-15

**Authors:** Hongbo Liu, Hao Li, Yuan Liang, Xinying Du, Chaojie Yang, Lang Yang, Jing Xie, Rongtao Zhao, Yigang Tong, Shaofu Qiu, Hongbin Song

**Affiliations:** 1 Academy of Military Medical Sciences, 27 Taiping Road, Haidian District, Beijing 100850, China; 2 The Centre for Infectious Disease Control, Chinese PLA Centre for Disease Control and Prevention, 20 Dongda Street, Fengtai District, Beijing 100071, China; 3 State Key Laboratory of Pathogen and Biosecurity, Beijing Institute of Microbiology and Epidemiology, 20 Dongda Street, Fengtai District, Beijing 100071, China; 4 Beijing Advanced Innovation Center for Soft Matter Science and Engineering (BAIC-SM), College of Life Science and Technology, Beijing University of Chemical Technology, Beijing 100029, China

**Keywords:** Antimicrobial resistance, Phage delivery system, CRISPR/Cas, Phage genome recombination, Phage therapy

## Abstract

Temperate phages integrated with clustered regularly interspaced short palindromic repeat (CRISPR)/Cas systems have been gaining attention as potential strategies for combating bacteria resistant to antimicrobials. To further advance this technology, phage recombination procedure should be improved, and the bactericidal effect should be examined in detail and compared with conventional lytic phage strategy. The possibility of the emergence of mutational resistance, a phenomenon commonly observed with lytic phage therapy, should be illustrated.

**Methods**: Here, we developed a novel one-step cloning method to fulfil the recombination of CRISPR/Cas9 system within the genome of a new isolated lysogenic *Escherichia coli* phage. Then, we proposed and developed a phage-delivered resistance eradication with subsequent antibiotic treatment (PRESA) strategy. The removal efficiency and antimicrobial effect of the plasmids were analysed. Long-term antimicrobial effect was evaluated by continued OD_600_ monitoring for 240 hours to illustrate the potential mutational resistance, compared with the lytic phage strategy. The treatment effect of PRESA was evaluated *in vivo* by determining bacterial loads in the skin and intestine of infected mice, in contrast with lytic phage therapy. Genome sequencing was performed to identify mutations in bacterial cells treated with phage strategies.

**Results**: Phage-delivered CRISPR targeting efficiently eradicated and blocked the transfer of the antibiotic resistance plasmid. PRESA decreased the bacterial load by over 6- and 5-logs *in vitro* and *in vivo*, respectively. Importantly, while lytic phages induced mutational phage resistance at 24 h *in vitro* and 48 hours *in vivo*, PRESA demonstrated a constant effect and revealed no resistant mutants. Genes involved in DNA mismatch repair were upregulated in cells undergoing Cas9-based plasmid cleavage, which may reduce the development of mutations.

**Conclusion**: The PRESA strategy for eradicating resistant bacteria showed high bactericidal efficacy and a sustained inhibition effect against resistant bacteria. By restoring the efficacy of low-cost antibiotics, PRESA could be developed as an efficient and economical therapy for infections of antibiotic resistant bacteria.

## Introduction

Bacterial antibiotic resistance has become a serious threat to human health 1-3. Superbugs, such as *bla*_NDM_- or *mcr*-possessing pathogens, have developed resistance against carbapenems or polymyxins, which are considered antibiotics of last resort 4-9. Unfortunately, the development of new classes of antibiotics has not kept pace with clinical demand. Thus, novel antimicrobial techniques defending against bacterial drug resistance are urgently needed.

Lytic phages are considered a promising strategy for combating antibiotic resistant bacteria as they are natural and effective predators of bacteria. The use of lytic phages against bacteria resistant to antimicrobials has revealed potent treatment effects 10, 11. However, phages that kill bacteria directly by cell lysis or cytotoxicity always result in bacterial phage-resistance. This resistance develops because of the emergence of mutant bacterial clones 10, 12. The phage-resistant mutants become predominant under the selection pressure of phage activity, which leads to a rapid loss of the bactericidal effectiveness of lytic phages 13-15. Thus, particular lytic phages cannot provide long-term and stable bactericidal effectiveness 16.

In recent years, temperate phage-delivered clustered regularly interspaced short palindromic repeat (CRISPR)/Cas (PDC) strategies have been developed to combat bacteria resistant to antimicrobials. CRISPR/Cas cassettes are integrated in the genome of temperate phages to eradicate resistant bacteria by lethal chromosomal DNA cleavage or resistance-encoding plasmid removal 17-20. Although the idea of combating antibiotic-resistant bacteria via RNA-guided nuclease targeting is intriguing, previous studies of chromosome targeting strategies have shown limited therapeutic effects; only a 10^2^ to 10^3^-fold decrease in bacterial load has been observed 17, 18. Essentially, modified temperate phages that cause lethal double strand breaks (DSBs) in the bacterial chromosome can be considered as a type of lytic phage. However, they are far less effective than native lytic phages since lytic phage therapies require no genome recombination process and have shown reliable therapeutic potential 15, 21. A resistance-encoding plasmid targeting strategy has been shown to sensitise resistant bacteria *in vitro* 20. However, whether this strategy can be utilised to eradicate resistant bacteria is yet to be evaluated, especially using *in vivo* assays.

Eradication of bacterial resistance can restore sensitivity to common antibiotics. In this study, we used a CRISPR/Cas9 system for targeting and eliminating resistance-encoding plasmids and presented a PDC-based strategy with potent antimicrobial efficacy. For efficient delivery, we designed a one-step engineering method to integrate the CRISPR/Cas9 system, capable of efficiently destroying NDM-1-encoding plasmids that mediate resistance against carbapenems within the genome of a novel temperate phage. The plasmid elimination and bacterial sensitising efficacy of PDC were then determined using both *in vitro* and *in vivo* assays. Next, the efficacy of this phage-delivered resistance eradication with subsequent antibiotic treatment (PRESA) strategy for eradicating resistant bacteria was evaluated. As PRESA targets resistant bacteria without phage-induced cell lysing activity, it has the potential to prevent the rapid development of bacterial phage resistance. Therefore, the long-term antimicrobial effectiveness of PRESA was observed *in vitro* and *in vivo* to determine whether new bacterial resistance against PRESA occurred during treatment. To highlight the value of PRESA, a lytic phage was examined in parallel as a direct killing strategy comparison. Finally, bacterial clones receiving PRESA or other parallel treatment strategies were analysed for the emergence of bacterial antibiotic or phage resistance-related mutations. By eradicating plasmid-encoding resistance and preventing the development of antibiotic or phage resistance, the efficacy of low-cost antibiotics can be restored for the treatment of bacterial infections. Thus, PRESA could be developed as a potential solution for the problem of bacterial antimicrobial resistance.

## Methods

### Plasmid and strain construction

Specific CRISPR plasmids were constructed based on pCas9 (plasmid 42876; Addgene, Cambridge, MA, USA). Specific spacer oligo pairs were synthesised and inserted into pCas9 according to the manufacturer's protocol. All spacers in the reengineered CRISPR systems were verified by sequencing with the spacer-F/R primers (for all primers see Table S1).

*E. coli* J53 pNDM-1 and *E. coli* J53 p*mcr-1* were generated by bacterial conjugation. The donor plasmids were obtained from clinical isolates. pEGFP_lac_-ndm was constructed from pEGFP-N1 (GenBank Accession #U55762; Clontech, Mountain View, CA, USA). The prokaryotic *lac* promoter was added to the upstream region of *EGFP*, and *bla*_NDM-1_ was amplified from pNDM-1 (primers NDM1001-F/R) and inserted into pEGFP-N1 between the *Xba*I and *Sal*I restriction sites. Recombinant plasmid pEGFP_lac_-ndm was transformed into *E. coli* DH5α competent cells to generate the fluorescent model strain *E. coli* DH5α pEGFP_lac_-ndm. The sucrose-induced suicide plasmid pST_K_ was constructed by inserting *sacB* into the *Nde*I restriction site in pUC-19 and integration of a kanamycin-selectable marker sequence between the *Sal*I and *Bam*HI sites in the same pUC19. The kanamycin-selectable marker was amplified using primers (N1Kana-F/R), containing the target-PAM sequence from *bla*_NDM-1_ at the 5′ end of primer N1Kana-F. Plasmids pUC_k_ and pUCtarget_k_ were constructed based on pUC-19, with the insertion of a kanamycin-selectable marker using primers Kana-F/R and N1kana-F/R, respectively. Resistant *E. coli* model strains used for the analysis of plasmid eradication by vB_Cas9 were constructed by transforming pUCtarget_k_ into wild-type *E. coli* strains.

### Determination of the plasmid-eradication effect

The PCR primers targeting pNDM-1 were ndm300-F/R and the primers for pMCR were clr-F/R. The relative fold change values of the plasmids in bacterial cells were determined using qPCR; 16S was used as the reference gene (primers 16s-F/R). Quantitative PCR was performed using primers ndm300-F/R with SYBR Premix Ex Taq (Tli RNase H Plus; Takara, Beijing, China) and a Bio-Rad qPCR instrument (Hercules, CA, USA). Amplification conditions were set according to the instructions provided with the qPCR reagents.

### Phage preparation and genome modification assays

Temperate phage vB_EcoM-IME365 was a novel lambda-like phage, which whole genome sequence length is 47.175 kb (Text S1). The host strain of this phage includes *E. coli* strains like MG1655, DH5α, J53. The entire length of lytic phage vB_Ecos-IME253 (referred to as vB_253) (GenBank Accession: KX130960) is 46.17 kb. It can infect and lyse the MG1655, DH5α, J53 strains. The methods for phage purification were similar to those in a previously reported study 20. Phage titre was determined using plaque-forming assays or qPCR with primers Phage200-F/R. Modification of the phage genome was performed using our one-step method. This method based on traditional bacterial homologous recombination method, but without introducing of the resistance-selective marker. First, the *E. coli* MG1655 strain was infected with temperate phage vB_365. Next, pKD46 and pST_k_ were transformed into lysogenic cells. The linear dsDNA donor was amplified and fusion PCR with primers phageup-F/R, spCas9-F/R and phagedown-F/R and purified using the Wizard PCR Preps DNA Purification System (Promega, Madison, WI, USA). Then, the *E. coli* MG1655 pKD46 pST_k_ prophage strain was grown in LB medium at 30 °C and 200 rpm, and the expression of the λ-Red system was induced by the addition of 0.02 mol/mL L-arabinose when the OD_600_ reached 0.1. When the OD_600_ reached 0.6, competent cells were obtained, and the dsDNA was electro-transformed. Transformant colonies were screened with LB agar containing ampicillin and sucrose, and then verified using PCR detection with primers phicas-F/R.

### Determination of the bacterial sensitising effect *in vitro*

*E. coli* containing the targeted plasmid was cultured at 30 °C until mid-exponential phase. The bacterial culture was then aliquoted into a 96-well plate (100 μL per well), and phages were added to each well at a MOI as indicated in each assay. Bacteria in the plate were incubated at 30 °C for 4 h and cells were cultured on LB medium plates to yield single colonies. Intracellular plasmids of bacterial colonies were determined by qPCR following the same method described above. The time course of plasmid eradication by the modified phage was determined by culturing resistant bacteria, adding the vB_Cas9 to bacterial liquid cultures for different time periods, and quantifying the relative fold change of the plasmid in the bacterial cells at the indicated time. In the PRESA strategy assays, cells were treated with phages together with kanamycin (25 μg/mL).

### *In vivo* assays

All animal experiments were supervised and approved by the Ethics Committee of Chinese PLA Centre for Disease Control and Prevention. All *in vivo* assays were conducted using 6- to 8-week-old female BALB/c mice. In this study, the mouse model used for superficial infection assays was modified from the thermal injury model of Stieritz and Holder 22. All mice were initially anesthetised with 4% pentobarbital sodium (4 µL/g of weight) by intraperitoneal injection. The fur on the mice backs was carefully shaved and removed with an 8% Na_2_S solution. Next, the denuded mice were sterilised. A 1.5 cm × 1.5 cm burn wound on mice backs was made by exposing this area at 95 °C for 10 s. Wounded skin was immediately challenged with inoculation of 10 µL PBS containing 10^5^ *E. coli* MG1655 pUCtarget_k_ cells. After 1 h, 20 µL of either vB_Cas9 or the wild-type phage solution (~10^7^ PFU) were subcutaneously injected under the infected skin. An additional 50 µL of the phage lysate was applied by direct spread on the wound. The wounded skin was covered with a moisturising dressing, and the activity of the mice was restricted to protect the wound. Kanamycin treatment was administered by tail vein injection. Wounded skin was homogenised to recover the colonised bacterial cells on MacConkey medium agar. Separated MG1655 cells were confirmed using 16S sequencing. *E. coli* clones were counted (by estimated CFUs) to calculate the bacterial load on burned skin tissue. A mouse model of the intestine was prepared by the administration of 50 µL of PBS containing ~10^5^ cells of* E. coli* MG1655 pUCtarget_k_ into the mouse intestine via an enema. Phages were also delivered by administration of 100 µL of a lysate containing ~10^7^ of either vB_Cas9 or the wild-type control. DNA was extracted from fresh stool samples using the QIAamp Fast DNA Stool Mini Kit (Qiagen, Hilden, Germany). Concentration of pUCtarget_k_ was quantified using qPCR with a standard curve as a reference. MG1655 cells in stool samples were isolated on MacConkey agar and identified using 16S sequencing. Number of colonies in each group were counted.

### Sequence analysis

In total, seven vB_253-resistant clones and four kanamycin-resistant clones were isolated from the *in vitro* experiment and subjected to genome sequencing, along with three clones isolated from the combined treatment group and one wildtype MG1655 strain. The genomic DNA of all MG1655 strains was extracted using the QIAamp DNA Mini Kit. A DNA library was constructed using the NEBNext Ultra II DNA Library Prep Kit for Illumina. Genome sequencing was performed with an Illumina MiSeq sequencing platform, and annotation was obtained using RAST. Sequence alignment analysis was performed using CLC Genomic Workbench 9.0 (CLC). The on-line version of the MG1655 genome was set as the reference (GenBank Accession: NC_000913.3). All reads of each clone were aligned with the reference sequence to determine the position and type of mutations and indicate gene annotations in the genome. Phage resistance-associated genes, including *ompA*,* ompC*,* ompF*,* phoE*,* lamB*,* fhuA*,* tonB*,* waaB,* and *btuB*, were screened.

### Transcription analysis of mismatch repair genes

A volume of 1 mL MG1655 pUCtarget_k_ cells (OD_600_ ≈ 0.3) was treated with the modified phage strategy (vB_Cas9) or lytic phage strategy (vB_253) separately (MOI ≈ 50). An equal amount of MG1655 cells was treated with kanamycin (final concentration 50 μg/mL); MG1655 pUCtarget_k_ cells were treated with saline as the blank control. Bacterial cells were collected after 24 h, and bacterial RNA was extracted using the TaKaRa MiniBEST Universal RNA Extraction Kit. cDNA was prepared using the PrimeScript RT reagent Kit with gDNA Eraser (TaKaRa). Following reverse transcription, the cDNA level of *mutS*, *mutT*, *polB* and *dnaE* was determined using relative quantitative PCR.

### Statistical analysis

Graphs were obtained using GraphPad Prism. Statistical analyses were performed with unpaired *t*-test. *P* < 0.05 was considered statistically significant. Error bars represent the standard deviation of at least three independent experiments.

## Results

### One-step integration of the CRISPR/Cas9 system into a lysogenic phage

**Figure 1:**
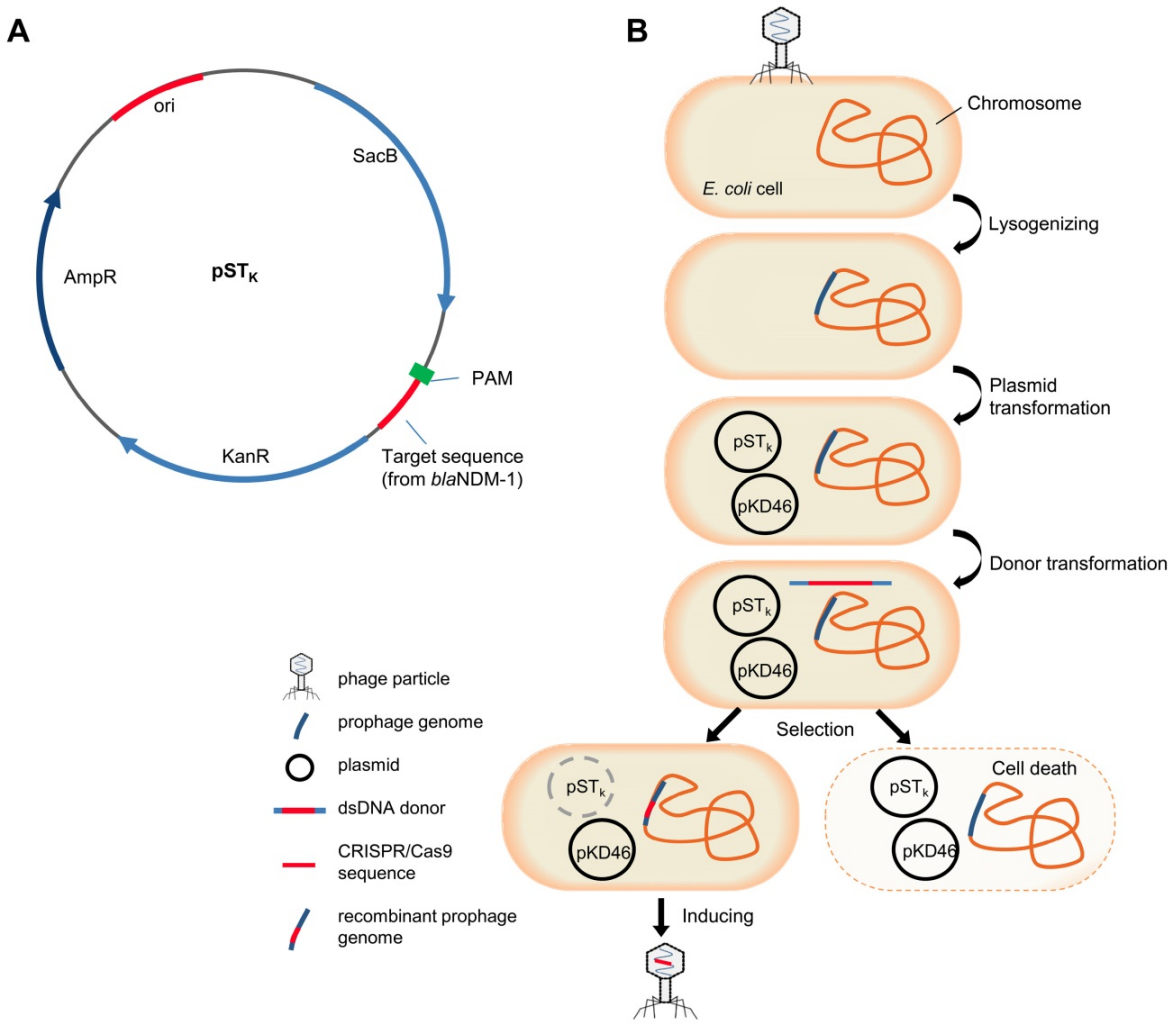
The scheme of suicide gene-based counter-selection system plasmid and temperate phage recombination procedure. (A) Target and PAM sequence of *bla*NDM-1 was integrated with the suicide gene *SacB* in plasmid pST_K_. (B) Integration of the CRISPR/Cas9 system within the temperate phage genome. MG1655 cells were lysogenised with the vB_365 prophage, and pKD46 and pST_k_ were subsequently transformed into this strain. The linear dsDNA recombination donor was prepared and delivered by electro-transformation. Transformants were screened on sucrose plates. Cells that recombined with CRISPR/Cas9 could survive in the presence of the activated suicide gene. vB_Cas9 was induced and harvested from the bacterial cells.

Plasmid pCas9-N1 possessing a CRISPR/Cas9 system targeting *bla*_NDM-1_ was constructed based on pCas9. Quantitative PCR results proved that target NDM-harbouring plasmids could be efficiently eradicated by pCas9-N1 in 8 h in* E. coli* cells, independent of plasmid copy number (Figure S1, S2). This highly functional CRISPR/Cas9 cassette was then inserted into temperate phage vB_EcoM-IME365 (vB_365). To integrate the CRISPR/Cas9 sequence of pCas9-N1 into the phage genome, we designed and implemented a novel seamless recombination procedure. First, we designed a counter-selection system and constructed plasmid pST_k_, which contained the sucrose-induced suicide gene *sacB* and a 30 bp target sequence (with PAM) from *bla*_NDM-1_ (Figure 1A). Subsequently, the vB_365 prophage, pST_k_ plasmid and pKD46 plasmid were successively introduced into the *E. coli* MG1655 host strain, providing the necessary enzymes for homologous recombination. Next, the dsDNA donor, containing the specific CRISPR/Cas9 cassette from pCas9-N1 and homologous arms, was transformed into the cells by electro-transformation. The CRISPR/Cas9 system integrated within the prophage genome could be directed to the target-PAM sequence and eliminate the suicide plasmid via an RNA-guided nuclease, enabling cell survival. In contrast, bacterial transformants that failed to integrate the CRISPR/Cas9 system were killed by the suicide gene (Figure 1B). CRISPR/Cas9-positive recombinants were further identified by PCR screening, which revealed a recombination rate of 28.1% (Figure S3). Finally, the genetically modified phage vB_EcoM-IME365_Cas9 (referred to as vB_Cas9) was induced from the recombinant host strain. The modified phage was shown to be CRISPR/Cas9-positive by PCR analysis (Figure S4). In addition, a 6.542 kb DNA fragment flanking the full length of the CRISPR/Cas9 sequence within the modified phage genome was examined to verify the integration of the specific CRISPR/Cas9 cassette (Figure S5). In comparison with previous methods (Table S2), our CRISPR/Cas9 integration process does not leave resistance marker residues in the phage particles. In addition, without deleting resistance selective markers, our method completes the genome recombination process in a single step, thereby shortening the procedure.

### Bacterial sensitisation efficacy

**Figure 2:**
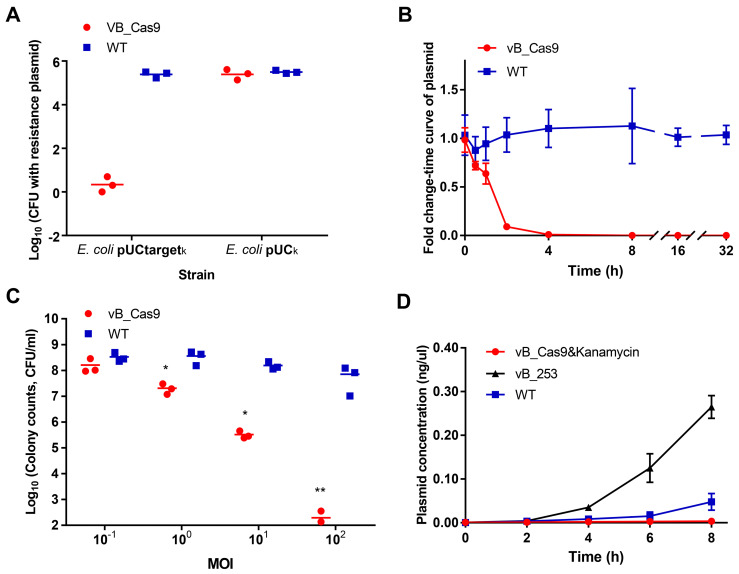
The phage delivered CRISPR/Cas9 was applied to eliminate resistance plasmids. Plasmids with or without the target site were treated with the modified vB_Cas9 phage or the wild-type temperate phage (WT) at a MOI of ~20 for 8 h. (A) Data represent the colony counts of resistance plasmid-possessing bacteria following different phage treatment (n = 3, mean ± SD). (B) Time-course analysis of the MG1655 pUCtarget_k_ strain treated with vB_Cas9 or the wild-type control. The relative fold change of pUCtarget_k_ copy number was determined at various time points using qPCR (n = 3, mean ± SD). (C) Kanamycin-resistant strains containing the targeted plasmid were cultured with vB_Cas9 or the wild-type control at various MOIs for 8 h. Surviving bacterial cells were determined by colony counts (n = 3, mean ± SD). (D) MG1655 pUCtarget_k_ cells was treated with combined use of modified phage and kanamycin, lytic vB_253 phage or wild-type vB_365 control. The concentration of plasmid in the bacterial supernatant was quantified by qPCR (n = 3, mean ± SD).

The sensitisation efficiency of the modified phage was determined *in vitro*. To verify the specific degradation of the target plasmid, we performed a plasmid curing assay by infecting *E. coli* MG1655 cells containing pUCtarget_k_ (with target sequence) or pUC_k_ (without target sequence) with modified phage vB_Cas9. Bacterial cells were continually recovered on LB agar and analysed for the presence of intracellular plasmids using colony PCR. The results indicated that vB_Cas9 decreased the number of resistance plasmid-possessing bacteria by over 5 logs (Figure 2A). Additionally, the efficiency of intracellular plasmid elimination was analysed by qPCR (Figure 2B). Nearly 99% of the intracellular resistance plasmids were eradicated in 4 h, over 99.99% of the plasmids were eradicated in 8 h and the elimination effect continued through 32 h. These results demonstrate the high efficacy of the Cas9 nuclease against resistance plasmids. Next, a combined strategy against resistant *E. coli*, involving phage-induced sensitisation and kanamycin treatment, was tested (Figure 2C). The bactericidal efficiency of this strategy increased with increasing multiplicity of infection (MOI); > 6-log reduction in colony forming units (CFUs) was detected at a MOI of 10^2^. In total, nine *E. coli* host strains were transformed with pUCtarget_k_ to test the plasmid elimination effect of the modified phage. The bacterial sensitisation effect was confirmed for all of these host strains. Furthermore, the combined strategy could block the spread of resistance by preventing the distribution of the responsible plasmids. Specifically, the transfer of pNDM-1 from *Acinetobacter* XM1750 to *E. coli* J53 was inhibited by the intervention of the modified phage. We further analysed the accumulation of resistance genes in the bacterial supernatant during the killing of the resistant strain. To highlight the advantages of PDC, the modified phage was compared with lytic *E. coli* phage clone vB_ 253. The bacterial supernatant of each group (5 μL) was filtered. Plasmid contained in the supernatant was transformed into *E. coli* DH5α competent cells. According to result of colony counts, the accumulation of resistance plasmids in the supernatant was slower in the modified phage group than in the lytic phage and control groups; the modified phage group yielded fewer transformant colonies compared with the lytic phage group at 6 and 8 h (Figure 2D; unpaired *t*-test, n = 3, *P* < 0.0001).

### PRESA sustained inhibition effect to the growth of antibiotic resistant bacteria *in vitro*

**Figure 3:**
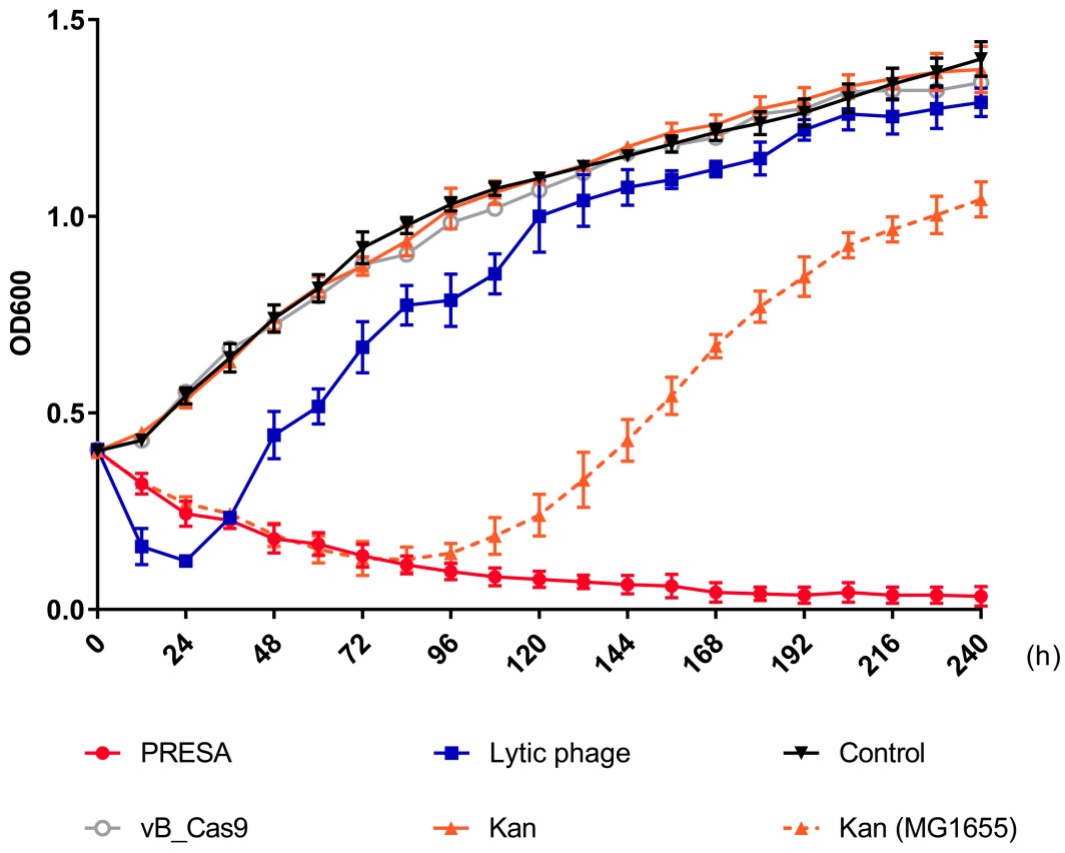
Solid curves represent the growth of the MG1655 pUCtarget_k_ strain treated with PRESA, lytic phage vB_253, kanamycin, vB_Cas9 and the PBS control. Phages added in each group were at a MOI of 10. The dashed curve represents the growth of MG1655 strain treated with kanamycin. Each dot represents the OD_600_ value (n = 3, mean ± SD) at the indicated time point relative to 0 h.

*E. coli* cells with plasmid pUCtargetk mediated Kanamycin-resistance were killed using PRESA. As mutational resistance can be triggered by phage or antibiotic strategies and compromise the antimicrobial effect, we determined the OD_600_ every 12 h to analyse the inhibition of bacterial growth using different strategies until 240 h (Figure 3). The modified phage and kanamycin (25 μg/mL) combination demonstrated continued efficacy against resistant strain *E. coli* MG1655 pUCtarget_k_. New resistance to PRESA was not observed until 240 h. In contrast, the *E. coli* vB_253 was used as a lytic phage strategy. The lytic phage resulted in a decrease in OD_600_ values during the initial 24 h but soon lost its inhibitory effect. The surviving bacterial cells in PRESA and lytic phage groups were separated at 96, 144, 192, and 240 h. A phage plaque test was used to confirm that the MG1655 cells in the vB_253 group had acquired phage-resistance after receiving the lytic phage strategy while cells in the combinatory treatment group had not (Figure S6). In addition, the wild-type MG1655 strain treated with kanamycin was used as a parallel experiment group, to determine induced kanamycin-resistance mutant. The OD_600_ results showed that bacterial growth was inhibited by kanamycin during the first 84 h, but subsequently, resistant mutants emerged and the OD_600_ value increased until the end of experiment. This result indicates that the combinatory strategy of PDC and kanamycin prevented both the rapid development of phage resistance and the emergence of kanamycin-resistant mutant clones.

### PRESA eradicated resistant *E. coli* in a mouse skin infection model

**Figure 4:**
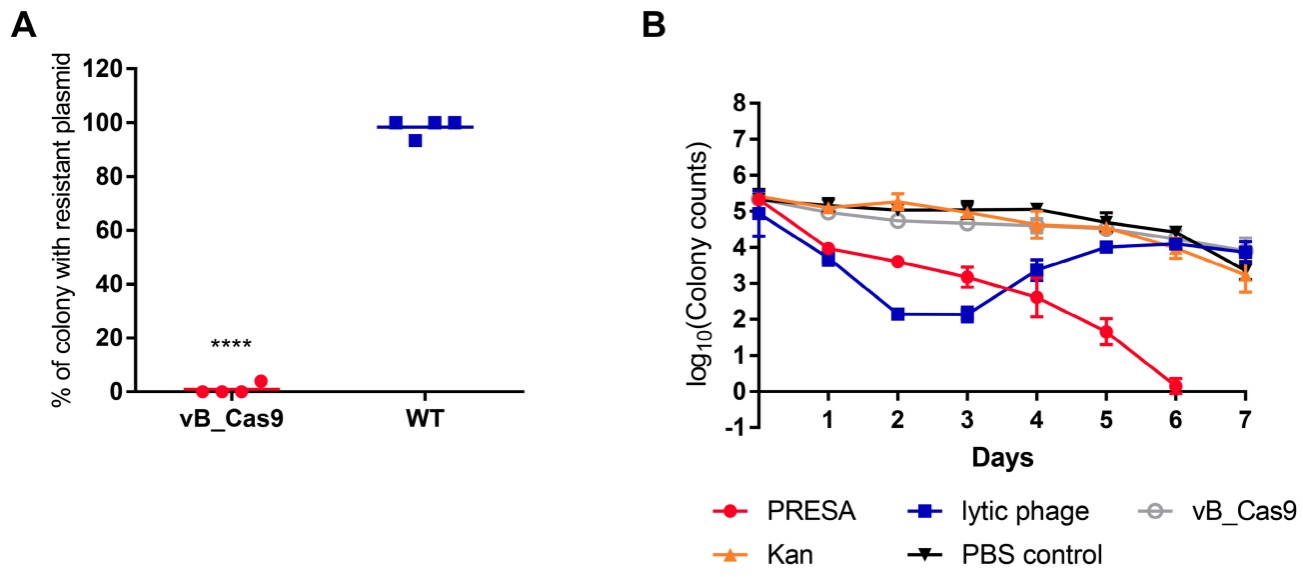
PRESA eradicated resistant *E. coli* in a mouse skin infection model. (A) The proportion of MG1655 cells containing pUCtarget_k_ in infected skin was determined 12 h after treatment with vB_Cas9 or WT temperate phage. Each value represents the percentage of plasmid-positive cells in each isolated mouse skin sample (n = 4, mean ± SD). (B) Infected mouse skin was treated with PRESA, lytic phage vB_253, kanamycin, vB_Cas9, or a PBS control. Data represent the logarithm value of bacterial cell counts per gram of skin (n = 3, mean ± SD).

We next investigated the therapeutic benefit of the bacteria-sensitising effect of the modified phage in a mouse skin infection model. As *E. coli* is a common species identified in burn wounds, we tested the sensitisation effect in a mouse model of infected skin. An area on the mouse back was depilated and superficially wounded. Next, the burned area was colonised with 10^7^ CFU of *E. coli MG1655* pUCtarget_k_. Then the burned area was treated with the modified phage or wild type vB_365. Then the sensitising effect of the modified phage on mouse skin was examined using PCR. Twelve hours after the modified phage treatment, only 6.67% bacterial cells isolated from the wounded skin were positive for the pUCtarget_k_, which was significantly lower than in the control group, i.e., 98.33% (unpaired *t*-test, *P* < 0.0001; Figure 4A). To demonstrate the advantages of the modified phage strategy, a combination of modified phage administration and kanamycin injection was used to kill MG1655 pUCtarget_k_ cells in mouse skin infection model, in comparison with the lytic phage strategy. The treatments were implemented every 24 h and the bacterial load in the infected skin was determined for the next seven days. The results showed that the lytic phage strategy decreased the bacterial load during the first two days (Figure 4B). However, the inhibition effect of the lytic phage ceased on the third day, and the bacterial load recovered from the fourth day. The developed lytic phage resistance was confirmed using a phage plaque test (Figure S7). The modified phage and kanamycin combination yielded a constant bacterial growth inhibition effect. New resistance to the combinatory treatment and recovery of bacterial growth were not observed.

### PRESA eradicated resistant *E. coli* in a mouse intestine infection model

**Figure 5:**
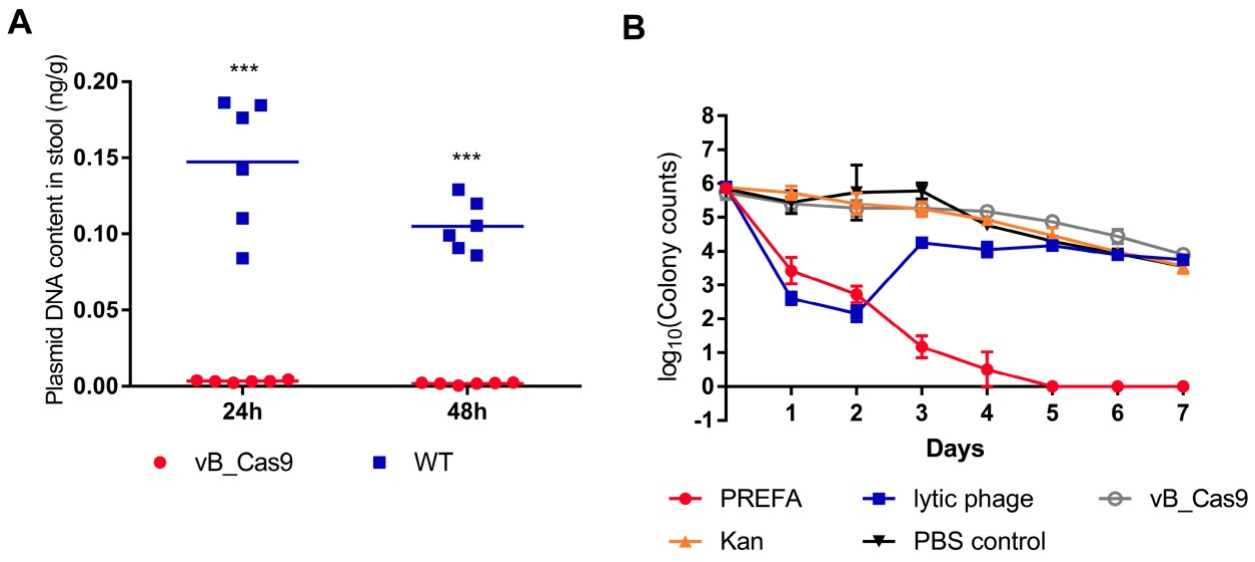
PRESA eradicated resistant *E. coli* in a mouse intestine infection model. (A) MG1655 pUCtarget_k_ cells in the mouse intestine were treated with vB_Cas9 or the WT temperate phage. Data represent the concentration of pUCtarget_k_ per gram of stool sample collected from each mouse (n = 6, mean ± SD). (B) The mouse intestine was treated with PRESA, lytic phage vB_253, kanamycin, vB_Cas9 or the PBS control. Data represent the logarithm value of bacterial cell counts per gram stool sample (n = 3, mean ± SD).

*E. coli* colonises the lower digestive tract and harbours resistance plasmids that can be transferred to *Enterobacteriaceae* strains. Accordingly, we tested the effectiveness of plasmid eradication using the phage delivered CRISPR/Cas9 system in the mouse intestine. The mouse intestine model was prepared by administration of an *E. coli* MG1655 pUCtarget_k_ inoculum into the mouse intestine. After 4 h, either vB_Cas9 or wild-type phage was applied to the mouse intestine by enema. Next, mouse stool samples were collected, and the concentration of pUCtarget_k_ in the stool samples was determined. Treatment with the modified phage resulted in a reduction in the target plasmid concentration in the stool samples to 3.45 ± 0.76 × 10^-3^ ng/g at 24 h and 1.79 ± 0.73 × 10^-3^ ng/g at 48 h; these concentrations were significantly different from the plasmid concentrations in the wild-type control-treated samples, i.e., 14.72 ± 4.27 × 10^-2^ ng/g at 24 h and 10.50 ± 1.68 × 10^-2^ ng/g at 48 h (Figure 5A; unpaired *t*-test, *P* < 0.0001). These results indicate that the phage delivered CRISPR/Cas9 system could effectively reduce antibiotic resistance gene levels in the mouse intestine. Next, different strategies were used to treat kanamycin-resistant *E. coli* MG1655 pUCtarget_k_ in a mouse intestine model (Figure 5B). Lytic phage vB_253 treatment reduced the *E. coli* cell count in stool samples on the first and second day. This inhibition effect was lost from the third day. However, treatment using the combinatory strategy showed persistent efficacy until the final day of the experiment.

### PRESA eradicated bacteria without the development of resistant mutant

**Figure 6:**
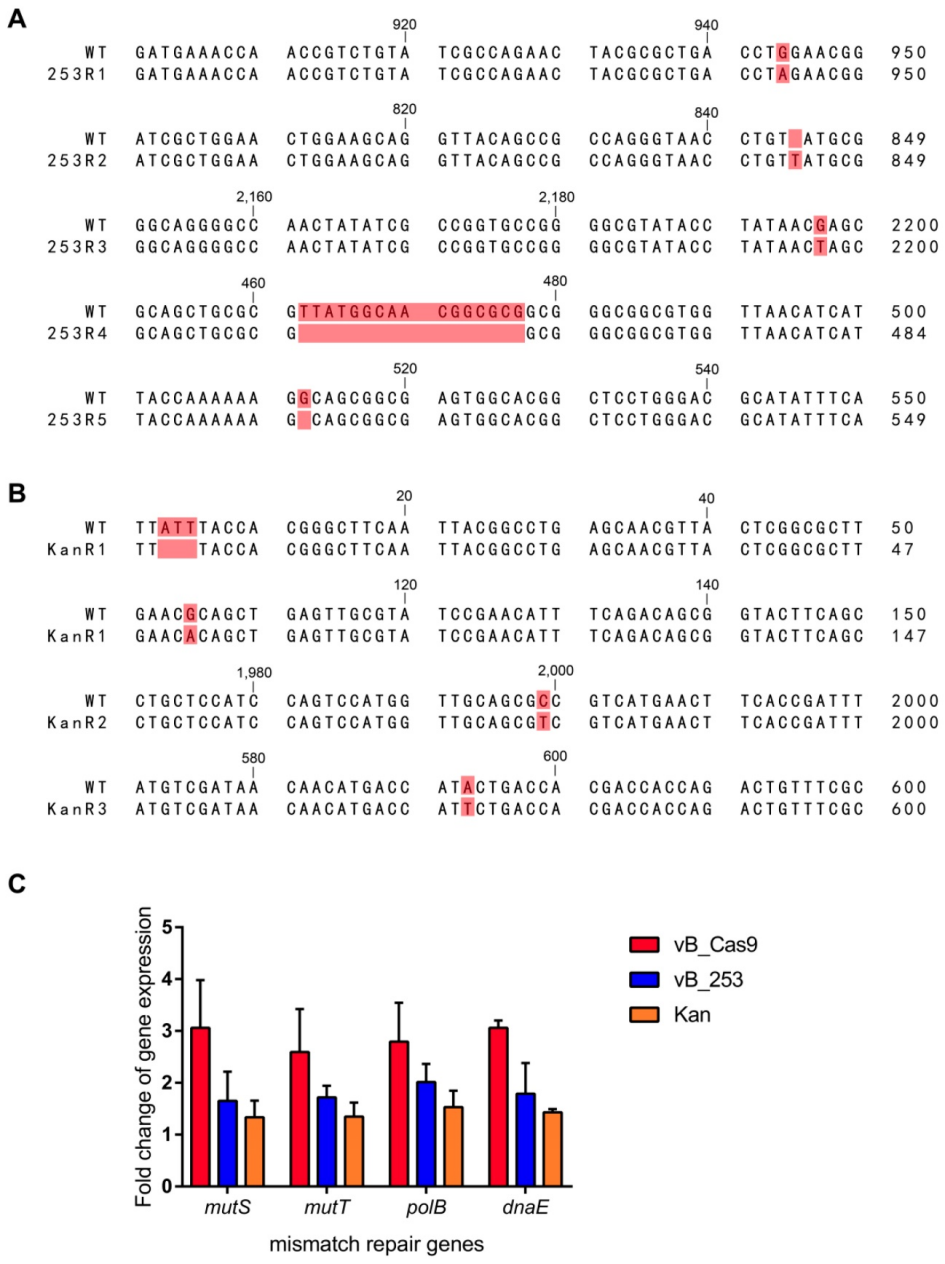
Sequence alignments of resistant mutant clones treated with wild-type MG1655, including clones with emerged resistance following treatment with the lytic phage (A) and with kanamycin (B). (C) Expression of genes involved in DNA mismatch repair (*mutS*, *mutT*, *polB* and *dnaE*; n = 3, mean ± SD).

Lytic phage-resistant *E. coli* clones were induced during treatment using phage vB_253. The emergence of phage resistance compromises the phage therapy efficacy. The vB_253-resistant MG1655 clones that emerged in the *in vitro* assay were isolated and subjected to genome sequence analysis. Five of the seven sequenced clones (253R1, 253R2, 253R3, 253R4, and 253R5) contained mutations in *fepA* (Figure 6A), including SNV (253R1: 943A>G, 253R3: 2197G>T), insertion (253R2: 844_845insT), and deletion (253R4: 462_477delTTATGGCAACGGCGCG, 253R5: 512delG). The *fepA* gene encodes the FepA protein, which is the ferric enterobactin outer membrane transporter involved in phage absorption as a recognition receptor 23. The kanamycin-resistant mutant showed a unique mutation in *fusA* (Figure 6B), which encodes protein chain elongation factor EF-G. Three of the four mutant clones (KanR1, KanR2, and KanR3) contained mutations in *fusA,* including SNV (KanR1: 105A>G, KanR2: 1979C>T, KanR3: 572T>A) and a deletion (KanR1: 4_6delATT). These phage- or kanamycin-resistance related mutations above, as well as other mutations in several common phage receptor genes and membrane protein genes (*lamb*, *ompA*, *ompC*, *ompF*, *phoE*, *phuA*, *tonB*, *waaB*, *btuB*), were not observed in the sequence of *E. coli* clones treated with PRESA. The expression of DNA mismatch repair genes at the mRNA level was examined using RT-qPCR (Figure 6C). Transcription of a number of genes (*mutS*, *mutT*, *polB* and *dnaE*) was upregulated in MG1655 cells receiving Cas9-based plasmid eradication, compared with phage- or kanamycin- resistant clones.

## Discussion

The concept of utilising genetic technology to combat antibiotic resistant bacteria has attracted substantial attention 24, 25. In this study, we evaluated a strategy for combating resistant bacteria using a combination of CRISPR-based sensitisation and conventional antibiotic treatment. This strategy restored the efficacy of low-cost drugs and efficiently eradicated resistant *E. coli* cells without the emergence of new resistance against PRESA.

Temperate *E. coli* phage was engineered using our novel method to integrate the CRISPR-Cas9 system within its genome. A negative screening system was designed based on combining the suicide gene *sacB* and the target sequence. This screening system was introduced into homologous recombination to replace the traditional screening system based on resistance markers 26. It guaranteed that temperate phages can integrate with a functional CRISPR/Cas9 system within one recombination step without any resistance residue in its genome. In addition, false positive recombination, such as mutations in the CRISPR/Cas9 cassette 18, could be precluded. Then the highly functional CRISPR/Cas9 system was efficiently delivered to the target and destroyed the bacterial resistance plasmid, yielding > 99.99% clearance rate of the target plasmid in 8 h. Plasmid removal not only eradicated the antibiotic resistance of *E. coli* cells, but also prevented the spread of bacterial antibiotic resistance. Plasmid-mediated horizontal gene transfer of drug-resistance was blocked by PDC. In contrast, lytic phages killed the bacterial cells but led to the dissemination of intracellular antibiotic resistance plasmids in the bacterial environment. These plasmids are possible to be taken up by co-harboured strains, resulting in the emergence of new resistant strains 27-31. Therefore, direct killing of resistant cells might increase the risk of horizontal transfer of resistance factors in bacteria-rich environments, such as the human intestine, hospital surfaces, and livestock farms 32, 33. Thus, plasmid targeting by PDC is not only expected to offer a novel alternative for resistant bacterial infections but will also aid in blocking the transfer of antibiotic resistance.

Owing to efficient target plasmid elimination and bacterial sensitisation, kanamycin sensitivity was restored and could be used to treat resistant *E. coli*. The PRESA strategy yielded a potent antimicrobial effect against resistant *E. coli* and the bacterial load decreased by 6 logs *in vitro* and 5 - 6 logs in the mouse skin or intestine infection models, showing a greater bactericidal effect than in previous studies 17, 18. Our results indicate that plasmid targeting strategy has the potential to facilitate antibiotic treatment of resistant bacteria in the future. Some antibiotic resistance genes are located in the bacterial chromosome, which is not suitable for using the PRESA strategy. However, according to previous studies, our modified phage can also be utilised to cleave chromosome-encoded antibiotic resistance genes, leading to bacterial cell death. There were concerns about the possibility that the temperate phage-based strategy could spread antibiotic resistance or virulence genes by phage delivery 34. In our assessment, the risk is low because when treating bacterial infections, the combined use of antibiotics can eradicate the strains receiving phage-induced transduction and sensitisation.

Most importantly, eradication of bacteria using PRESA did not lead to mutational resistance, which is always observed using lytic phage treatment. In addition to acquired antibiotic resistance mediated by mobile plasmids, emergence of mutational antimicrobial resistance is another threat during bacterial infection treatment. Direct killing strategies are more likely to result in the selection of resistant mutants 34. Various studies have demonstrated that the efficacy of phage antimicrobials is rapidly compromised because of the development of bacterial resistance against the phage 35-38. In most cases, phage resistance arises owing to changes in receptors on the bacterial cell membrane, resulting in the absence or modification of phage receptors 15. Selection pressure exerted by lytic phages leads to the predominance of resistant mutants and the recovery of bacterial growth 39. Our results demonstrate that new bacterial resistance against lytic phage vB_253 rapidly developed by 12 h *in vitro*. Genome sequence analysis revealed that the occurrence of mutations in the *fepA* gene was closely related to this phage resistance. The outer membrane protein, FepA, participates in the adsorption of ferric enterobactin and also functions as the receptor of some phages 23, 40. However, PRESA exhibited persistent efficacy against antibiotic resistant bacteria. No mutations were found in *fepA* or other phage receptor-associated genes. In fact, our engineered temperate phage did not kill bacterial cells through repeated lytic life cycles but efficiently disarmed bacterial cells following infection. Thus, temperate phages carrying the CRISPR/Cas9 system do not trigger the selection of phage resistance and can sustain longer effectiveness compared with lytic phages.

Antibiotic often lost sensitivity because of the occurrence of mutational antibiotic resistance 41. Our results indicated that even the sensitive MG1655 strain developed new resistance against kanamycin after treating with kanamycin for three days *in vitro*. Sequence analysis discovered mutations in the *fusA* gene in the genome of kanamycin-resistant mutant clones. The *fusA* gene encodes the translation elongation factor EF-G; mutations in this gene are responsible for the development of kanamycin-resistance 42, 43. Surprisingly, our results suggest that the PRESA strategy prevents the formation of mutational kanamycin-resistance. This phenomenon might be associated with the nuclease activity of CRISPR/Cas9. It has been reported that double stand breaks in bacterial DNA result in the activation of the DNA repair mechanisms 44. Indeed, RT-qPCR analysis showed that the transcription of DNA mismatch repair-related genes was increased after *E. coli* cells underwent targeted plasmid cleavage. The rise of gene mutations associated with drug resistance could be suppressed due to the activation of DNA mismatch repair mechanisms. Further research is still needed to reveal the underlying mechanism of this phenomenon.

The PRESA strategy exhibited a potent effect against resistant bacteria and prevented the re-emergence of new antibiotic or phage resistance. Further development and application of this strategy could provide a solution for the antibiotic resistance crisis. Also, this research illustrated the benefit of the combined use of novel phage strategy and conventional antibiotics. Previous studies have shown that phage-resistance often leads to a decrease of bacterial fitness or antibiotic resistance 36, 37. These findings provide increasing evidence that phage-based therapy may become a solution for bacterial resistance. A limitation that needs to be addressed is the narrow host range of phage. Utilising broad-host range phages or a cocktail strategy may solve this problem. Advances in nano-materials may also provide new solutions to CRISPR/Cas9 delivery 45, 46. With better delivery systems, a CRISPR-based bacterial sensitisation strategy could be extensively applied in clinical treatment, and well-established antibiotic therapies could become effective once again in treating drug-resistant infections.

## Conclusions

In this study, we demonstrated that the PRESA strategy effectively eradicated resistant bacteria by restoring the efficacy of conventional antibiotics. This strategy shows higher efficacy than previously reported CRISPR-based antimicrobials. Importantly, unlike the lytic phage strategy, PRESA exhibited a continuous inhibitory effect against resistant bacteria, without the emergence of new mutational resistance. Thus, the efficacy of low-cost antibiotics could be restored in bacterial infection treatment. Finally, we believe that PRESA can be further developed as a promising therapy to address the antibiotic resistance issue.
