# Supplementary material for: Phage-delivered sensitisation with subsequent antibiotic treatment reveals sustained effect against antimicrobial resistant bacteria
Source: Theranostics. 2020 May 15;10(14):6310–21. doi: 10.7150/thno.42573 (PMC7255016; doi:10.7150/thno.42573)
Supplement: Supplementary file 1 — Supplementary figures and tables. [file thnov10p6310s1.pdf]

Supplementary materials

Phage-delivered sensitisation with subsequent antibiotic treatment reveals sustained effect against antimicrobial resistant bacteria

Hongbo Liu[1,2,†], Hao Li[3,†], Yuan Liang[2,†], Xinying Du[2,†], Chaojie Yang[2], Lang Yang[1], Jing Xie[2], Rongtao Zhao[2], Yigang Tong[4,*], Shaofu Qiu[2,*], Hongbin Song[1,2,*]

[1]Academy of Military Medical Sciences, 27 Taiping Road, Haidian District, Beijing 100850, China;

[2]The Centre for Infectious Disease Control, Chinese PLA Centre for Disease Control and Prevention, 20 Dongda Street, Fengtai District, Beijing 100071, China;

[3]State Key Laboratory of Pathogen and Biosecurity, Beijing Institute of Microbiology and Epidemiology, 20 Dongda Street, Fengtai District, Beijing 100071, China

[4]Beijing Advanced Innovation Center for Soft Matter Science and Engineering (BAIC-SM), College of Life Science and Technology, Beijing University of Chemical Technology, Beijing 100029, China

[†]These authors contributed equally to this article.

[*]Corresponding Authors: hongbinsong@263.net (H.S.); qiushf0613@hotmail.com (S.Q.); tong.yigang@gmail.com (Y.T.).

Figure S1. Eradication of *bla*$_{NDM-1}$ possessing plasmids using pCas9-N1

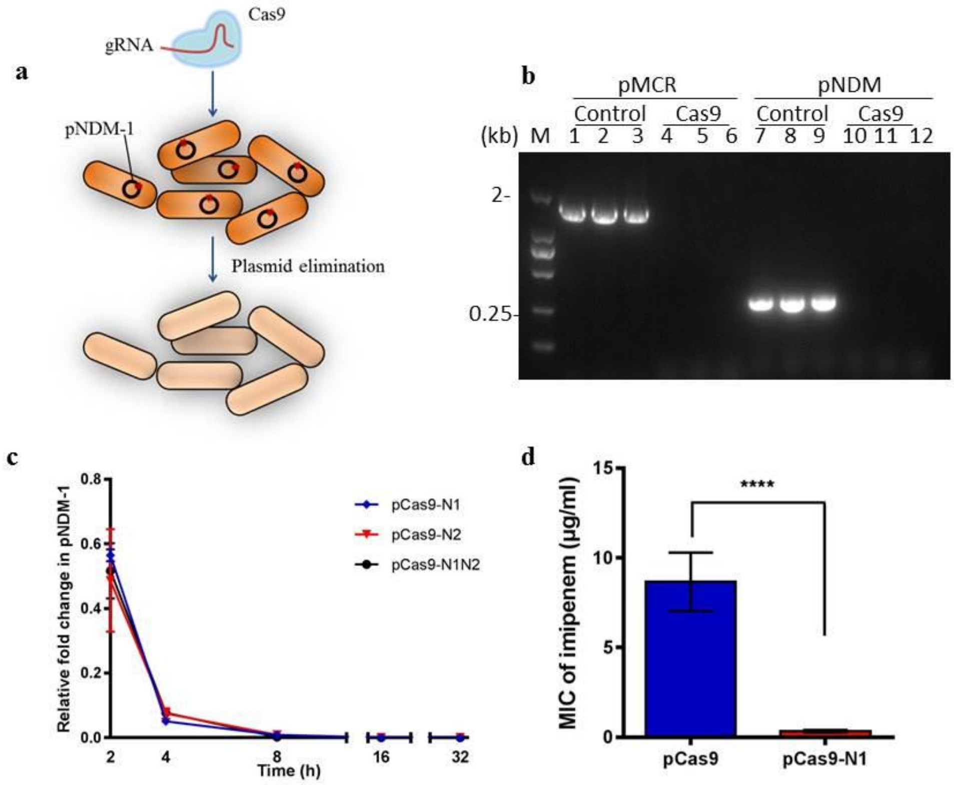

Sensitizing effect to antimicrobial resistant bacteria by CRISPR/Cas9 plasmid was determined *in vitro*. (a) The specific CRISPR/Cas9 plasmid was transformed into resistant *E.coli* J53 cells. (b) The specific CRISPR/Cas9 plasmids were delivered into J53 cells to target pNDM and pMCR. PCR detection of transformed colonies indicated that guided Cas9 enzyme could digest these resistant plasmids separately, controlled by pCas9 blank vector. (c) J53 pNDM-1 was sensitized using CRISPR/Cas9 plasmids targeting one site in *bla*$_{NDM-1}$ (pCas9-N1, pCas9-N2) or two sites (pCas9-N1N2). The relative fold change of pNDM-1 copies were determined at various time points by qPCR (n = 3, mean ± SD). Results revealed that targeted resistant plasmid was eliminated by over 99.9% after 8h. (d) Antimicrobial susceptibility test showed that MIC of imipenem of J53 pNDM-1 cells was obviously decreased after transformation of pCas9-N1 (unpaired t-test, n=3, $p<0.001$).

Figure S2. Eliminate high-copy NDM-1 plasmid using pCas9-N1

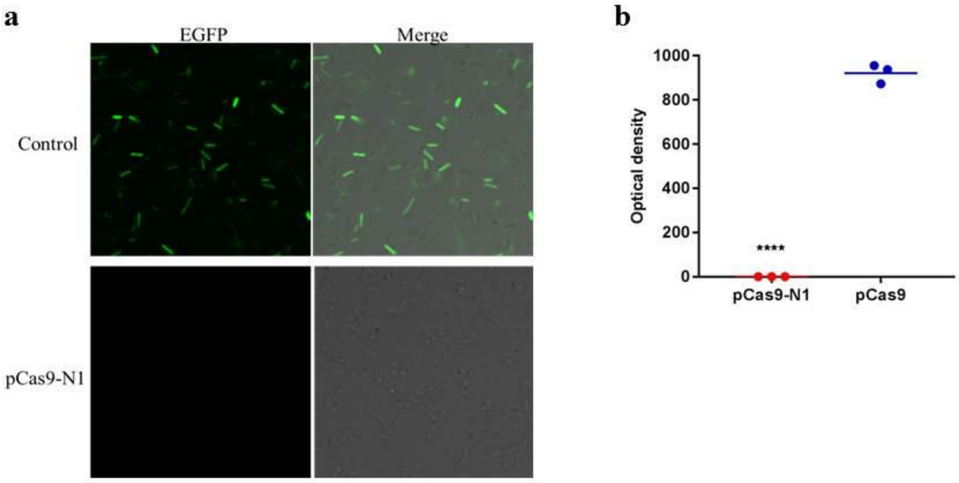

High-copy plasmids pEGFP-ndm co-expressed *bla*$_{\mathrm{NDM-1}}$ and *EGFP*. (a) The *E. coli* DH5α pEGFP-ndm were transformed with pCas9-N1 and pCas9 control separately. EGFP signals were scanned by fluorescence confocal microscopy. EGFP signals were significantly quenched in pCas9-N1 group compared to those of the control. (b) The integrated optical fluorescent density decreased from 921.5 ± 24.99 to 0 (unpaired *t*-test, $P < 0.0001$, $n = 3$), representing a decrease in *bla*$_{\mathrm{NDM-1}}$ of greater than 99.9%.

Figure S3. PCR screening of dsDNA transformant colonies

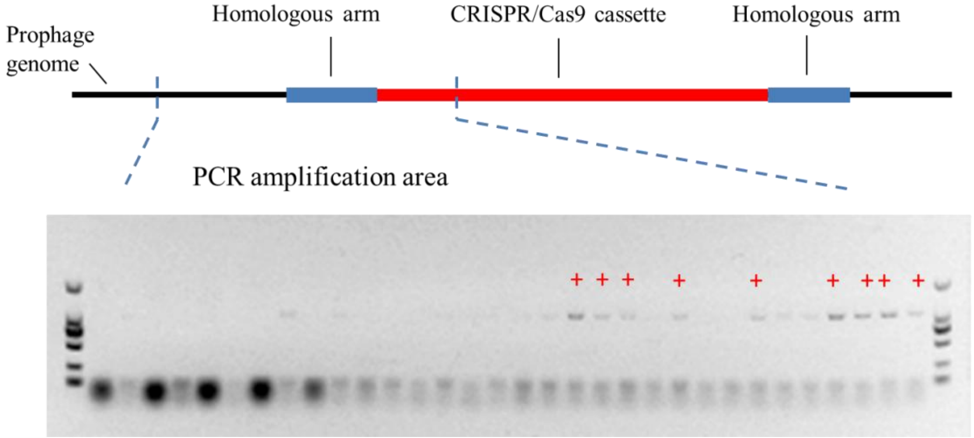

Colonies transformed with dsDNA were screened by PCR. The amplification flanked the upstream homologous arm. Using this screening strategy, colonies did not complete the recombination would show negative results in PCR screening, and transformants recombined with CRISPR/Cas9 cassette in prophage genome could be identified. Nine out of thirty-two colonies were identified as positive recombinant transformants by PCR detection. Positive rate was 28.1%.

Figure S4. PCR examination to engineered genome of modified phage

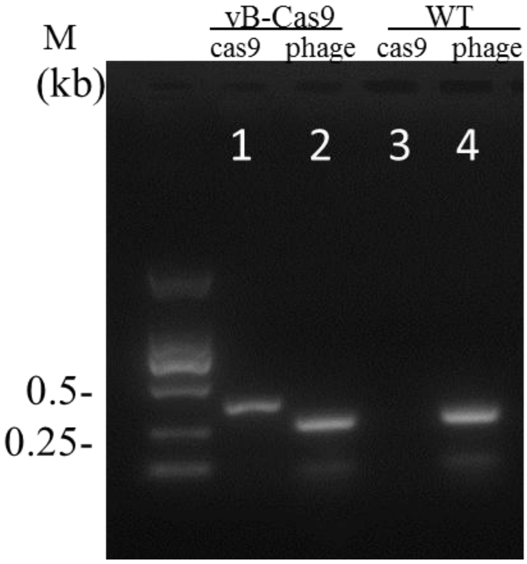

Genome-engineered phage was examined by PCR, compared with control. The vB_Cas9 phage group (lane1, 2) showed positive for both cas9 gene and phage genome, while wild-type control (lane3, 4) was negative for cas9 sequence.

Figure S5. Verification the integration of CRISPR/Cas9 in phage genome by PCR sequencing

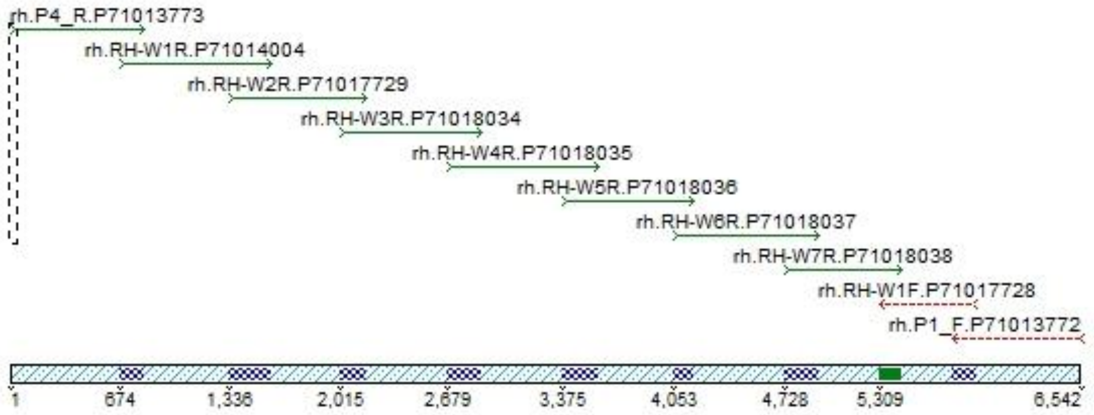

PCR sequencing of a 6.542 kb fragment in modified phage genome containing full length of CRISPR/Cas9 cassette (5.081 kb), also flanking full sequence of dsDNA donor for phage recombination. This whole fragment was assembled by results of ten sequencing reactions, and its sequence was displayed as below.

Assembled sequencing results:
ATTATATTTAGCCCTACCAGTGACAATAGCGTAACGGAAAACCTCGCCGCAT
CTTCTGCGGGCTTTGTTGGCTCGCTCCATTGCACCGCGATCTTCAAATCTGC
GGATTACTTCCAGCAGTTGCATCGGCTCAATATCCTGAATTTCAAGGCCGCC
GATGATAGGTAAAATGTCGTCATCAAACATTTTTGCAAGTTCAGTTGCATAG
CCTACTGACCAGACTTGCTTCTTGTGCTCGTACCATTCCTTGTAAATCGCAC
TAAAGGAATTGTTGTTAGACGAAGCCTTTTTCGCTTTTACCGGATCGATGCC
AACCGAGATGTCTTTCCTCGCGGTCCATGCTTTATCTCTTGCCTCCTGCAAA
GTCATTAGCGGATATTTTCCTACGGTCAGGATTTTCTCCTTACCGTCAATCTT
GTAGCGAAGCTGCCATACCTTTTTCCCTGACACAGGGACATAAAGGTACAG
GCCATTACCATCGAGTAGGCGGTATGGTTTTTCTTTCGGCTTTGCTGCTTCA
ATCTGCTTAACGGTGAGCATGGGTAAAAATCCGGTGGGTAAAATTATTTTAT
CCACTTTTTACCCGTCATGGAGTGCGGCTGTCAACGATCTGACGCGAACCA
TTACGAACTGTGAACACACTACTCTTCTTTTGCCTATTATAACATGAAAACG
CTTTAACAGAAAGAATAGGAAGGTATCCGACTGCTGGTATTAACCCTCTTTC
TCAAGTTATCATCGGCAATGTTGAATGGAGTCCATTCAAAACAGCATAGCTC
TAAAACATGGAACTCAACAAGTCTCAGTGTGCTGAAGTTTTGGGACCATTC
AAAACAGCATAGCTCTAAAACGCTCAGCATCAATGCAGCGGCTAATGCGGT
GTTTTGGGACCATTCAAAACAGCATAGCTCTAAAACCTCGTAGACTATTTTT
GTCTAAAAAATTTCGTAATCGCACTATTTGTCTCAGCTAGACTTCAGTCTTG
AAAAGCCCCTGTATTACTGCATTTATTAAGAGTATTATACCATATTTTAGTTA
TTAAGAAATAATCTTCATCTAAAATATACTTCAGTCACCTCCTAGCTGACTC
AAATCAATGCGTGTTTCATAAAGACCAGTGATGGATTGATGGATAAGAGTG
GCATCTAAAACTTCTTTTGTAGACGTATATCGTTTACGATCAATTGTTGTATC
AAAATATTTAAAAGCAGCGGGAGCTCCAAGATTCGTCAACGTAAATAAATG
AATAATATTTTCTGCTTGTTCACGTATTGGTTTGTCTCTATGTTTGTTATATGC

ACTAAGAACTTTATCTAAATTGGCATCTGCTAAAATAACACGCTTAGAAAAT
TCACTGATTTGCTCAATAATCTCATCTAAATAATGCTTATGCTGCTCCACAAA
CAATTGTTTTTGTTCGTTATCTTCTGGACTACCCTTCAACTTTTCATAATGAC
TAGCTAAATATAAAAAATTCACATATTTGCTTGGCAGAGCCAGCTCATTTCC
TTTTTGTAATTCTCCGGCACTAGCCAGCATCCGTTTACGACCGTTTTCTAAC
TCAAAAAGACTATATTTAGGTAGTTTAATGATTAAGTCTTTTTTAACTTCCTT
ATATCCTTTAGCTTCTAAAAAGTCAATCGGATTTTTTTCAAAGGAACTTCTT
TCCATAATTGTGATCCCTAGTAACTCTTTAACGGATTTTAACTTCTTCGATTT
CCCTTTTTCCACCTTAGCAACCACTAGGACTGAATAAGCTACCGTTGGACTA
TCAAAACCACCATATTTTTTTGGATCCCAGTCTTTTTTACGAGCAATAAGCT
TGTCCGAATTTCTTTTTGGTAAAATTGACTCCTTGGAGAATCCGCCTGTCTG
TACTTCTGTTTTCTTGACAATATTGACTTGGGGCATGGACAATACTTTGCGC
ACTGTGGCAAAATCTCGCCCTTTATCCCAGACAATTTCTCCAGTTTCCCCAT
TAGTTTCGATTAGAGGGCGTTTGCGAATCTCTCCATTTGCAAGTGTAATTTC
TGTTTTGAAGAAGTTCATGATATTAGAGTAAAAGAAATATTTTGCGGTTGCT
TTGCCTATTTCTTGCTCAGACTTAGCAATCATTTTACGAACATCATAAACTTT
ATAATCACCATAGACAAACTCCGATTCAAGTTTTGGATATTTCTTAATCAAA
GCAGTTCCAACGACGGCATTTAGATACGCATCATGGGCATGATGGTAATTGT
TAATCTCACGTACTTTATAGAATTGGAAATCTTTTCGGAAGTCAGAAACTAA
TTTAGATTTTAAGGTAATCACTTTAACCTCTCGAATAAGTTTATCATTTTCAT
CGTATTTAGTATTCATGCGACTATCCAAAATTTGTGCCACATGCTTAGTGATT
TGGCGAGTTTCAACCAATTGGCGTTTGATAAAACCAGCTTTATCAAGTTCA
CTCAAACCTCCACGTTCAGCTTTCGTTAAATTATCAAACTTACGTTGAGTGA
TTAACTTGGCGTTTAGAAGTTGTCTCCAATAGTTTTTCATCTTTTTGACTACT
TCTTCACTTGGAACGTTATCCGATTTACCACGATTTTTATCAGAACGCGTTA
AGACCTTATTGTCTATTGAATCGTCTTTAAGGAAACTTTGTGGAACAATGTG
ATCGACATCATAATCACTTAAACGATTAATATCTAATTCTTGGTCCACATACA
TGTCTCTTCCATTTTGGAGATAATAGAGATAGAGCTTTTCATTTTGCAATTGA
GTATTTTCAACAGGATGCTCTTTAAGAATCTGACTTCCTAATTCTTTGATACC
TTCTTCGATTCGTTTCATACGCTCTCGCGAATTTTTCTGGCCCTTTTGAGTTG
TCTGATTTTCACGTGCCATTTCAATAACGATATTTTCTGGCTTATGCCGCCCC
ATTACTTTGACCAATTCATCAACAACTTTTACAGTCTGTAAAATACCTTTTTT
AATAGCAGGGCTACCAGCTAAATTTGCAATATGTTCATGTAAACTATCGCCT
TGTCCAGACACTTGTGCTTTTTGAATGTCTTCTTTAAATGTCAAACTATCAT
CATGGATCAGCTGCATAAAATTGCGATTGGCAAAACCATCTGATTTCAAAA
AATCTAATATTGTTTTGCCAGATTGCTTATCCCTAATACCATTAATCAATTTTC
GAGACAAACGTCCCCAACCAGTATAACGGCGACGTTTAAGCTGTTTCATCA
CCTTATCATCAAAGAGGTGAGCATATGTTTTAAGTCTTTCCTCAATCATCTCC
CTATCTTCAAATAAGGTCAATGTTAAAACAATATCCTCTAAGATATCTTCATT
TTCTTCATTATCCAAAAAATCTTTATCTTTAATAATTTTTAGCAAATCATGGTA
GGTACCTAATGAAGCATTAAATCTATCTTCAACTCCTGAAATTTCAACACTA
TCAAAACATTCTATTTTTTTGAAATAATCTTCTTTTAATTGCTTAACGGTTAC
TTTTCGATTTGTTTTGAAGAGTAAATCAACAATGGCTTTCTTCTGTTCACCT
GAAAGAAATGCTGGTTTTCGCATTCCTTCAGTAACATATTTGACCTTTGTCA

ATTCGTTATAAACCGTAAAATACTCATAAAGCAAACTATGTTTTGGTAGTAC
TTTTTCATTTGGAAGATTTTTATCAAAGTTTGTCATGCGTTCAATAAATGATT
GAGCTGAAGCACCTTTATCGACAACTTCTTCAAAATTCCATGGGGTAATTGT
TTCTTCAGACTTCCGAGTCATCCATGCAAAACGACTATTGCCACGCGCCAA
TGGACCAACATAATAAGGAATTCGAAAAGTCAAGATTTTTTCAATCTTCTCA
CGATTGTCTTTTAAAAATGGATAAAAGTCTTCTTGTCTTCTCAAAATAGCAT
GCAGCTCACCCAAGTGAATTTGATGGGGAATAGAGCCGTTGTCAAAGGTCC
GTTGCTTGCGCAGCAAATCTTCACGATTTAGTTTCACCAATAATTCCTCAGT
ACCATCCATTTTTTCTAAAATTGGTTTGATAAATTTATAAAATTCTTCTTGGCT
AGCTCCCCCATCAATATAACCTGCATATCCGTTTTTTGATTGATCAAAAAAG
ATTTCTTTATACTTTTCTGGAAGTTGTTGTCGAACTAAAGCTTTTAAAAGAG
TCAAGTCTTGATGATGTTCATCGTAGCGTTTAATCATTGAAGCTGATAGGGG
AGCCTTAGTTATTTCAGTATTTACTCTTAGGATATCTGAAAGTAAAATAGCAT
CTGATAAATTCTTAGCTGCCAAAAACAAATCAGCATATTGATCTCCAATTTG
CGCCAATAAATTATCTAAATCATCATCGTAAGTATCTTTTGAAAGCTGTAATT
TAGCATCTTCTGCCAAATCAAAATTTGATTTAAAATTAGGGGTCAAACCCAA
TGACAAAGCAATGAGATTCCCAAATAAGCCATTTTTCTTCTCACCGGGGAG
CTGAGCAATGAGATTTTCTAATCGTCTTGATTTACTCAATCGTGCAGAAAGA
ATCGCTTTAGCATCTACTCCACTTGCGTTAATAGGGTTTTCTTCAAATAATTG
ATTGTAGGTTTGTACCAACTGGATAAATAGTTTGTCCACATCACTATTATCAG
GATTTAAATCTCCCTCAATCAAAAAATGACCACGAAACTTAATCATATGCGC
TAAGGCCAAATAGATTAAGCGCAAATCCGCTTTATCAGTAGAATCTACCAAT
TTTTTTCGCAGATGATAGATAGTTGGATATTTCTCATGATAAGCAACTTCATC
TACTATATTTCCAAAAATAGGATGACGTTCATGCTTCTTGTCTTCTTCCACCA
AAAAAGACTCTTCAAGTCGATGAAAGAAACTATCATCTACTTTCGCCATCT
CATTTGAAAAAATCTCCTGTAGATAACAAATACGATTCTTCCGACGTGTATA
CCTTCTACGAGCTGTCCGTTTGAGACGAGTCGCTTCCGCTGTCTCTCCACT
GTCAAATAAAAGAGCCCCTATAAGATTTTTTTTGATACTGTGGCGGTCTGTA
TTTCCCAGAACCTTGAACTTTTTAGACGGAACCTTATATTCATCAGTGATCA
CCGCCCATCCGACGCTATTTGTGCCGATATCTAAGCCTATTGAGTATTTCTTA
TCCATTTTTGCCTCCTAAAATAAAAAGTTTAAATTAAATCCATAATGAGTTTG
ATGATTTCAATAATAGTTTTAATGACCTCCGAAATTAGTTTAATATGCTTTAAT
TTTTCTTTTTCAAAATATCTCTTCAAAAAATATTACCCAATACTTAATAATAAA
TAGATTATAACACAAAATTCTTTTAAAAAGTAGTTTATTTTGTTATCATTCTAT
AGTATTAAGTATTGTTTTATGGCTGATAAATTTCTTTGAATTTCTCCTTGATTA
TTTGTTATAAAAGTTATAAAATAATCTTGTTGGAACCATTCAAAACAGCATA
GCAAGTTAAAATAAGGCTAGTCCGTTATCAACTTGAAAAAGTGGCACCGAG
TCGGTGCTTTTTTTGATACTTCTATTCTACTCTGACTGCAAACCAAAAAAAC
AAGCGCTTTCAAAACGCTTGTTTTATCATTTTTAGGGAAATTAATCTCTTAAT
CCTTTTATCATTCTACATTTAGGCGCTGCCATCTTGCTAAACCTACTAAGCTC
CACAGGATGATTTCGTAGGGCCCTCATCGCCATTGCTCCCCAAATACAAAA
CCAATTTCAGCCAGTGCCTCGTCCATTTTTTCGATGAACTCCGGCACCATCT
CGTCAAAACTCGCCATGTACTTTTCATTCCGCTCAATCACGACATAATGCAG
GCCTTCACGCTTCATGCGCGGGTCATAGTTGGCAAAGTACCAGGCATCTTT

TCGCGTCACCCACATGCTGTACTGCACCTGGGCCATGTAAGCCGATTTTATT
GCCTCGAAACCACCGAGCCGGAATTTCATGAAATCCCGGGAGGTAAACGG
GCATTTCAGTTCAAGGCCGTTGCCGTCACTGCATAAACCATCGGGAGAGCA
GGCGGTGCGCATATTTTCGTCGCGATAGATGATCGGGGATTCAGTAATATTC
ACGCCGGAAGTGAATTCAAACAGGGTTCTGGCGTCGTTCTCGTACTGTTTT
CCCCAGGCCAGCGCCTTAGCATTAACTTCCGGAGCCACACCGGTGCAAAC
CTCAGCCAGCAGGGTGTGGAAGTAGGACATTTTCATGTCAGGCCACTTCTT
TCCTGAGCGGGGCTTTGCTATCACGTTGTGAACTTCTGAAGCGGTGATGAC
GCCGAGCCGTAATTTGTGCCATGCATCATCCCCCTGTTCGACAGCTCTCACG
TCGATCCCGGTACGCTGCAGGATAATGTCCGGTGTCATGCTGCCACCTTCTG
CTCAGTGGCTTTCTGTTTCAGGAATCCAAGAGCTTTCACTGCTTCGGCCTG
TGTCAGTTCTGACGATGCGCGAATGTCGCGGCGAAATATCTGGGAACAGAG
CGGCAATAAGTCGTCATCCCA

Figure S6. Phage plaque test of MG1655 cells separated from phage treatments during 240 h observation

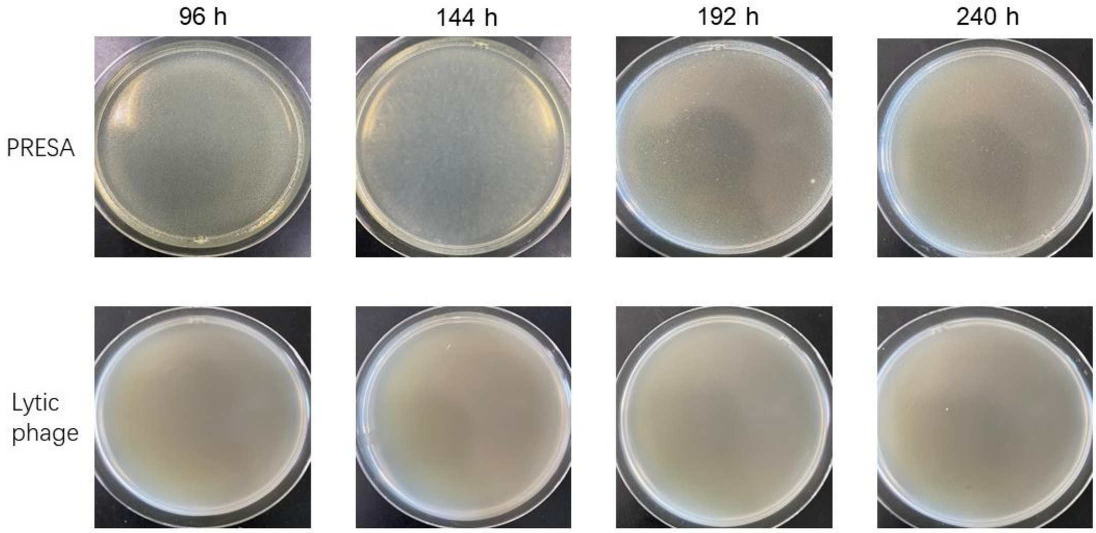

Host strain of phage plaque test were arranged using bacterial cells separating from the 240-hour in-vitro surveillance in different time points, to demonstrate the development of resistance of corresponding phages. Photos above represents the result of vB_253 plaque, which host strain were from the lytic phage treatment strategy. No forming plaque were found in this test. Photos below represents the result of SSC induced vB_Cas9 plaques, which host strain were from the vB_Cas9&Kan group. Even the rarely remaining cells in 192-hour treatment were also sensitive to phage infection.

Figure S7. Phage plaque test of MG1655 cells separated from phage treatments in mice skin.

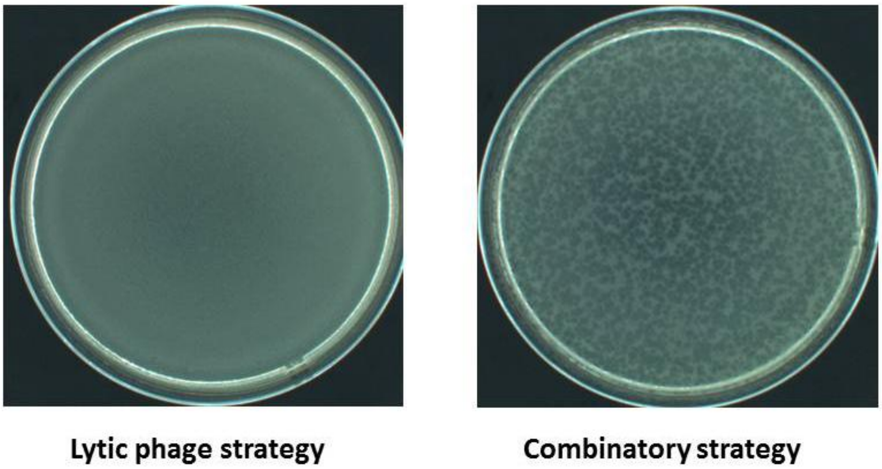

Phage plaque test were arranged using bacterial cells separating from the mice skin model in day 6, to demonstrate the development of resistance of corresponding phages in vivo. Left photo represent the result of vB_253 plaque, with host strain from the lytic phage strategy treatment. No forming plaque were found in this test, indicating the developed resistance to lytic phage of separated bacteria. Right photo represent the result of SSC induced vB_Cas9 plaques, which host strain were from the vB_Cas9&Kan group. The remaining bacteria were also sensitive to phage infection.

Figure S8. Phage plaque test of MG1655 cells separated from phage treatments in mice intestine.

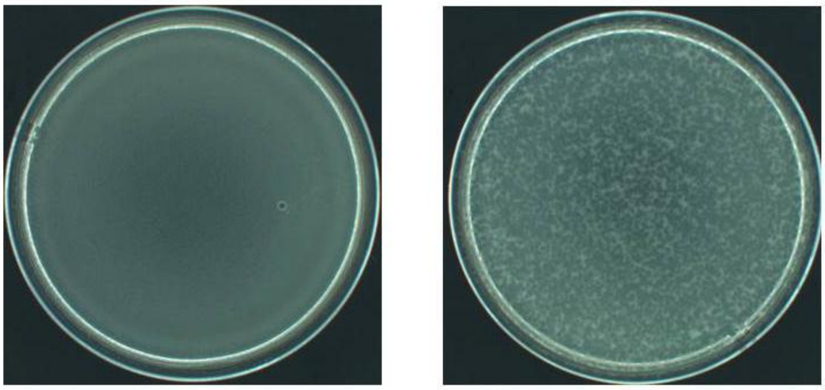

Lytic phage strategy   Combinatory strategy

Phage plaque test were arranged using bacterial cells separating from the mice intestine in day 7, to demonstrate the development of resistance of corresponding phages in vivo. Left photo represent the result of vB_253 plaque, whose host strain were from the lytic phage strategy treatment. No forming plaque were observed, indicating the developed resistance to lytic phage of separated bacteria. Right photo represent the result of SSC induced vB_Cas9 plaques, which host strain were from the vB_Cas9&Kan group. The remaining bacteria showed no phage resistance.

Table S1. Primers in this research

Primers	Sequence (5'-3')	Use
ndm300-F	GGCGGAATGGCTCATCACGA	detection of *bla*NDM-1
ndm300-R	CGCAACACAGCCTGACTTTC	detection of *bla*NDM-1
NDM1001-F	CGGTCGACCACCTCATGTTTG AATTCGC	detection of *bla*NDM-1
NDM1001-R	CCCTCTAGACTCTGTCACATC GAAATCGC	detection of *bla*NDM-1
16s-F	AGAGTTTGATCCTGGCTCAG	determination of reference gene
16s-R	CTGCTGCCTCCCGTAGGAGT	determination of reference gene
spacer-F	AACACGCATTGATTTGAG	detection of CRISPR loci
spacer-R	ATAGGAAGGTATCCGACT	detection of CRISPR loci
N1Kana-F	GTCGACACCGCATTAGCCGC TGCATTGATGCTGAGCAGGG CGCAAGGGCTGCTAAAG	amplification of KanR gene with target and PAM sequence
N1Kana-R	GGATCCTCAGAAGAACTCGT CAAGAAGGC	amplification of KanR gene with target and PAM sequence
Kana-F	AGCGTCGACTCAGGGCGCAA GGGCTGCTAAAG	amplification of KanR gene
Kana-R	CGCGGATCCTCAGAAGAACT CGTCAAGAAGGC	amplification of KanR gene
kana200-F	GCCCTCTGGTAAGGTTGG	determination of KanR gene
kana200-R	GCCGATTGTCTGTTGTGCC	determination of KanR gene
phageup-F	GCCTTGAACTGAAATGCCCG	amplification of upstream homologous arm
phageup-R	ATGATTTCGTAGGGCCCTCAT CGCCATTGCTCCCCAA	amplification of upstream homologous arm
spCas9-F	ATGGCGATGAGGGCCCTACG AAATCATCCTGTGGA	amplification of CRISPR/Cas9 cassette
spCas9-R	AACCATTACGAACTGTGAAC ACACTACTCTTCTTTTGC	amplification of CRISPR/Cas9 cassette
phagedown-F	AGAGTAGTGTGTTCACAGTT CGTAATGGTT	amplification of downstream homologous arm
phagedown-R	TTTCCTCGCGGTCCATGC	amplification of downstream homologous arm
phicas-F	CTGCGTTACCGAGTGGAT	confirm the recombination

		of CRISPR/Cas9 within the temperate phage
phicas-R	AAGGCTAGTCCGTTATCAA	confirm the recombination of CRISPR/Cas9 within the temperate phage
Cas350-F	GCCGTCGTTGGAACTGCTTT	GCCGTCGTTGGAACTG CTTT
Cas350-R	TCCTTGGAGAATCCGCCTGT	TCCTTGGAGAATCCGC CTGT
Phage200-F	ggaccagcatacgatctgccgt	detection of the temperate phage
Phage200-R	caggcaaagtccgtggctgat	detection of the temperate phage

Table S2. Comparison of techniques for construction of phage-delivered CRISPR/Cas9 system

Study	This study	Previous study(*13*)	Previous study(*12*)	Previous study(*15*)
Phage name	vB_EcoM_IME365	M13	ΦNM1	λ
Method for integration of CRISPR/Cas9	Homologous recombination	Phagemid construction	Phagemid construction	Homologous recombination
Method for Recombinant screening	Suicide gene based counter-selection, avoid resistant marker	Resistant marker based selection	Resistant marker based selection	Resistant marker based selection
Resistance residue	none	Remained in phage particle	Remained in phage particle	Deleted by another Flippase recombination

Text S1. Whole genome sequence of vB_365

>Seq1 [organism=Escherichia virus Lambda-like phage vB_EcoM_IME365],
chromosomeTAAGCATCAGTGAAACCTCGCCGCTTGCTCTGCTGGTTCAATG
GAAATAACCAGGTCTTTGTCGCCTAACTGCCAGATAATTCCCTGTTCTTCTT
CGTCCCCTGTCATTGCCTCCACATAAGCCATAAGGTAATTCATCATGATGTTT
ATGCCGTCGACTCCTTCGCTTTCTGCATCTTCCATGAGGTCAGAGAAACGC
TCGATGTAGTCTTCTTCTGCGCTCATAGCTATTTCCTCAGGTTGAACATTCGC
AAGGTAATGCGTCGTCATCGGCAAACATGTCTATTTGCAGAGCTCGCTCAG
CAACTGCTTGCTTAGCAATACTCCAGAATTTTCCAGATGGGACGTGTTCATT
TGGCTGAATTCCAAGTCTTTCCATTTCGGCAAATAACCCCTCCCTTTCCTCC
AAATAAACTGCGCCAGCTTCATCCTTGTGAATTGCATATCCGATTTCCTCTT
CCGCAGCTTTCGCCTCATTCCAGATGTCTTTACGAAGCACATAAACGCAGT
ACCAGTGTTGCCATCCTGCCTTTAAACAGCCAATGCAATTTGCGTGCTTAAA
TCTCGAATAAGCCATTGGGCGGTTAATGCCAATTTCTTCCGTAGAGTGGATT
GTTCTTTGCGGCCATAGGGCAATCGGATAGTCAGTTTTATAACCTCGTTCAG
CCATAACACCCACGCGGCGCTGAACTCGATGCATTTCATTTCTGTCAAATCC
GTAGTAGTAGATATTTTCCTTTTGGCTATCATTACGCTCCAGCCACAGATCAA
ACGGTCCTGTCTTAAGCCTGTTGGTGCATAGAATTTGACCTGATCCAACTTT
CCATGCCCTCGCGTCAAGGCAAACAGATATTGGAGTAGCGTCTTTCCACCT
TTCATGGTTGGCATACGTAACATCTATCCCTAAATAACTTGATATCTCATCCTT
AAATCTCTTGATGTCCTCGTCTTCTACTGTGCTACTGATGTCGTGGTTAAGC
AGGATGACATTTTCTCTTCCATATTTTCTGACCACCTCTATCGCGACAATTGC
CGAGGAGTGGCCTCCGCTATACAAAACTACGTGCTTCATGCCCATACCCTCT
GTTGATACTTACTCCTACCCTTTGGCTCGCTGGCGTATTCTGGAAGCAACGC
GGAAACCACCCATAACCGAGGGTCTGCGCTTAGCGTTCGTTGAGTTTTTAC
GTTGCGGGATTGGTAGGCGGTGATTAACTGAGTTGCTTCTTCGTTGGTGAG
GTCGTGATGGTGAAACCATGTGAGCTTCAATCGTACCTCCTGTCAGTGAAC
CTGACGCTCTGACCGATAGCCCAGGCAGTCGTATACTCTATGAGGCTCGCC
ATGCGCCCCACGCTCATCTGCGCGCTGCTTTCGCGAATGTTGACGTATTCGC
CTTCAAGCCCGGGCAAAACATCAGCTTCCTGCTTTGTTGCCACTGCATGAC
CGCTGATCAACAAAACCTTCCATTGTTCTGGTTTTAACCATTTATCGCGCCA
CTGAACTTGCCTGGCGATATCTGCGACCATAGCGTGAAATTTTGCGTTCTGG
TCAAGGTTGCGCTTGTAGTCAGTAATGCGGATGGTAACTGGCTTGTCTTTAT
CGAGTGGTGTTGCGAGGATGGCGTTGATTGCGGCTTGCTGTTGTTGCTTAG
TTCGGAGGAAGATTGTTTGCTTCATCGTTACCTCAACTCACAAAACGCCAC
GCCACTTTTGCTACGACAACAGGCATAACACCGATAATCACCCACAGGAAA
ATGCTACCGAAAAGCACACCAACCAAGTCTTTACCTTCGCCTACCAGCCGG
ACAAAACTGCTGGCAACCACAATGAACGTCGCCACCATCCACATAGCACC
GAGAATCCTCAATGCAGAAAAAATCAACTCAACCACGATTTACTCTCCCCC
AAATAAAAAGGCCTGCGATTACCAGCAGGCCTGTTATTAGCTCAGTGATGT
AGATGGTCATCTTTTAACTCCATATACCGCCAATACCCGTTTCATCGCGGCA
CTCTGGCGACACTCCTTAAAAATTAGGTTCGTGCTCATCTTTCCTTCCCGTT
CTTCCTTGGTAGCAAACCGGTAATACACCGTTCGCCAGACCTTACCTTCGAT
AGCCAGAAGACCTGCCCGTGCCATTTTAGCCGCAGCCTGATTTATGCTGGTT

ACCGTTGCGCCTGTTACCGCGGCAACGTCCTGCGCACAGAATTTCTTATGA
GTCCCCAGGTAATGAATAATTGCCTCTTTGCCCGTCATACCCTTGCTCCTTTC
AGCCCAAACTTAGCTTTGATTTCTGCGATCTTCGCCAGAGCCTGTGCACGA
TTTAGAGGTCTACCGCCCATGACAGGAAGTTGTTTTACTGGTTCAGGTATAG
CCTCACCACGGTTAATTCGTGCGGTCATACAGGACAGTTCATCGGCAGCCT
TGCGCCGTAATTCCGCGTCAGTCAACGCATTGGCCCGCATGTTCTGATACA
GGTTGGTAACCAGCCAGTAGTGCGCGTTTGATTTCCACGGATAAGACTCTG
CATCCGGATACAGCCCGCGCTTCCGGCAATACTCGTAAACCATATCAACCA
GCTCGCTGGCGTTTGGCAGCCCGGCGGTAACGGATGCTTCTTCCCGGCACC
AGGCGACAAACTGCCCGGGTGATGGCAGGAATGGTCGATTCTGCCGACGG
GCTACGCGCATTCCAGCGTTAACCTGTTCCATTGTGGTGATCCCGTTTTCCC
GAAAAGCCAGCACCCACTGGCGGCGGATTTCGTTCAGTTCATTCTGGTCAC
GGTTAGCCAGGCTCGCCGGGAAAGTTGCCAGTAACTGGCTGAACACACCG
TTGATGATCTGCGCTACCTGCTGTACCTGCGGCTTTTCGTCGTACTGTTCCG
GCATGTTGTTGGCGATCCGACGCATCTGCTCACGGTCAAAGTTAACCATCT
GTGCGGCGATGTTTTTCATAAATCCACCCCGTAAATCCAGTCAGTGTTTGTC
AGGTCGAGTTTTGGTTTGCTGGCTATCATGCCTGCCTGTTGCTTGTTACGGT
TGATTTCGAGTTGGGTCCACTTATCGCGGAGTTTGGCCGGGCTCAGCACGT
TACCGGACCAGAAGTTGTCCTGGCATGCCCAGCGGAACAGCACGCACATG
TCGCGGTGGTTACGTCCGTCACGTTCACGCATCAGGCGGATATCGTTAGCC
CACCCAGCAAAATTCGGTTTTCTGGCTGATGGTGCGATAGTCTTCACCATGT
CAAACATCCACTCTGCGGCGGTCAGGTCTTCTGCTGTCCCCCACTTGCTGC
CGCTCTGAATTGCAGCATCCGGTTTCACCACAGAAAGGTCGTTTTCTGGCT
GGTCAGAGGATTCGCCAGAATTCTCTGACGAATAATCTTTTCTTTTTTCTTT
TGTAATAGTGTCTTTTGTGTCCCCCTGTTTTGAGGGATAGCAATCCCCCAAT
TTGAGGGATGTTTTATCCCTCGTTTTAGGGGATTTTCCCTCGTTTTGAGGGA
TGCACCATTCTGAGATGTTTTTATTTGGTCCAAACATGCCGCCTTGCTGCTT
GATAATATTCATTCTGACGAGTTCTAACTTGGCTTCATTGCACCGTTTGACG
GGTAACTTTGTAATCTCGCTAAGTTGAGAATCGGTGATTCTGTCCATTGGTT
TATTCCACCCATAGGTTTTACGCAGAATGGCAAGCAGCACTTTAAACTGTC
GCTTGGTCAGATCTGCGCCCGAATAAGCCTCAAGCAGCATATTTGATAGTCT
GGCGTAACCATCATCGAGATCTGCCACATTACGCTCCTGTCCGGCAAAGTT
ACCTCTGCCGAAGTTGAGTATTTTTGCTGTATTTGTCATAATGACTCCTGTTG
ATAGATCCAGTAATGACCTCAGAACTCCATCTGGATTTGTTCAGAACGCTCG
GTTGCCGCCGGGCGTTTTTTATTGGTGAGAATCGCAGCAACTTGTCGCGCCA
ATCGAGCCATGTCGTCGTCAACGACCCCCCATTCAAGAACAGCAAGCAGC
ATTGAGAACTTTGGAATCCAGTCCCTCTTCCACCTGCTGATCTGCGACTTAT
CAACGCCCACAGCTTCCGCTGTCTTCTCAGTTCCAAGCATTGCGATTTTGTT
AAGCAACGCACTCTCGATTCGTAGAGCCTCGTTGCGTTTGTTTGCACGAAC
CATATGTAAGTATTTCCTTAGATAACAATTGATTGAATGTATGCAAATAAATG
CATACACCATAGGTGTGGTTTAATTTGATGCCCTTTTTCAGGGCTGGGATGT
GTAAGAGCGGGAATGTCTTAAGCGGCTTTACCGCGTTTAGTTCCGTACTGT
AACCAAACCGGATCACAGTTAAGCGCCATAGCAATCTCAAACAAGAAGCG
CGGTCGCTTGGTTACTCCAGCTTCAATCAGTTGAATTGATTGCTGTTTAACA

CCGGCTTTGGTTGCCAGTTCGGTTTGCGTCATTTTAACGCAATTCGCCTCT
TCTTGAGGCGTTCAGAAAGAGTTTGCATATCGCCTCCATCAACAAACTTTC
TTGTATTTTCATACAATGTATCTTGTTTGTCAAATACAGTTTTTCTTGTGAAG
ATTGGGGGGTAAATAACAGAGGTGGCTTATGAGTATTTCTTCCAGGGTAA
AAAGCAAAAGAATTCAGCTTGGACTTAACCAAGCTGAACTTGCTCAAAAG
GTGGGGACTACCCAGCAGTCTATAGAGCAGCTCGAAAACGGTAAAACTAA
GCGACCACGCTTTTTACCAGAACTTGCGTCAGCTCTTGGCGTAAGTGTTGA
CTGGCTGCTCAATGGCACCTCTGATTCGAATGTTAGATTTGTTGGGCACGTT
GAGCCCAAAGGGAAATATCCATTGATTAGCATGGTTAGAGCTGGTTCGTGG
TGTGAAGCTTGTGAACCCTACGATATCAAGGACATTGATGAATGGTATGAC
AGTGACGTTAACTTATTAGGCGATGGATTCTGGCTGAAGGTTGAAGGTGAT
TCCATGACCTCACCTGTAGGTCAAAGCATCCCTGAAGGTCATATGGTGTTAG
TAGATACTGGACGCGAGCCAGTGAATGGAAGCCTTGTTGTAGCCAAACTGA
CTGACGCGAACGAAGCAACATTCAAGAAACTGGTTATAGATGGCGGGCAG
AAGTACCTGAAAGGCCTGAATCCTTCATGGCCTATGACTCCTATCAACGGG
AACTGCAAGATTATCGGTGTTGTCGTGGAAGCGAGGGTAAAATTCGTATGA
TCAGGATTGCGGCGCTACTCTCAATACTCTTAACTACCAGCGCCAATTCTGA
ATGCTGGATTGTCACAAACCTGCACGGGTACGGGGCAATGAATGGCGATCG
TTACGAGTTTACAAAAGACAGCACGGAAGATTCCGTTTTCCACGTAACAAT
AAATGGCGATAAATCATCAGTTTATGAATCAGTTTCTGGCGTCTATCCAGAG
ATGAAATACACTGCTTTGTCATCGAACACTATGGTAGGAGAATACCAGTCTG
GAGGAGGAATAACCGTTGAAACCTGGTCAATCACTACAAACAAAAAAGCT
CTTTACTCCAAAGTAATGAACATCCCAGGTATGCAACAACTTACATCAACC
AAATCCTTTGTTGGTGATGTAGTCGGAACCTGCAACCAGTAATCCCCACCT
CAATCTCGATAACCAAAAAACAAACTATTTTCCGTTTAAAAACAATGAAGT
TTGTTTTTCATGCCCCTTTTTTACAATGTTTCTTGTTTACAACATACAATCTTT
CTTGTAATTTTAAGCCATCAGCAGGACGCACTGACCACCATTGAAGGTGAT
GCTCTTAAAAATTAAGCCCTGAAGAAGGGCAGCATTCAAAGCAGAAGGCT
TTGGGGTGTGTGATACGAAACGAAGCATTGGCCGGAAGTGCGAATCCGGA
TTAGCTGCAAATGAGCCAATCGTGGGGTGTTTTCGTTCAGGACTACGACTC
ACACACACCACCAAAGCTAACTGACAAGAGAATCCAGATGGATGCACAAA
CACGCCGCCGCGAACGTCGCGCAGAGAAACAGGCTCAATGGAAAGCAGC
AAATCCCCTGTTGGTTGGGGTAAGCGCAAAACCAGTTAACCGCCCTATTCT
CTCGCTGAATCGCAAACCGAAATCACGAGTAGAAAGCGCACTGAATCCGAT
AGACCTTACGGTGCTGGCTGAATACCACGAACAGATTGAAAGCAACCTGC
AACGTATTGAGCGCAAGAATCAGCGCACATGGTACAGCAAGCCTGGCGAA
CGCGGCATAACATGCAGAGGACGCCAGAAAATTAAAGGTAAATCTATATCA
CTTATTTAGAAAATGCAGATTTAGGGAACAGATAGGAGGCGTTACACCTATG
GCATCTCATCCTATGGTTAGAAGGTGGTGCAAATCCTTCGTATTGAAGTATG
GATTTCACAGAAGATTCATAGCATTGAGCGCAAAGATAGTGCATTGGCTGA
CCGGTATTTGCCGATTTTTTGAGACGATAAACTACCGTAGCAACAGTAGGTG
TATACATCTCATAGTTTTTCTTTTCCTCTTCCCACTTAGAAGCTCGATTTATCT
TTTCTTCAAGCTCAATAATCTTATCCTTAGAAATCATCAAAAGCTCATTAAGT
GACATTTGCTGCTGTTGGGCATCCATGAGCTTATCGACAAGTTCGTATGTTT

TTTCTTTTACTGAGTAGTCTATTTGCATTTTCTGGATTTCCTTTACTGCGCCA
ACAGCACTCATCAGAGCACCTCCGGCACCAGAAACTGCATCTGTAATCCTA
CTTATTATTCCTTTTTCATCAGACATATAAATCACTCTCTTACTGTAGGGGTA
AGAGGATTTTACTATTTTTCTCGCTGTAGGGGTACACGAGAACCACCGAGC
CTGATGTGGTTAAAAGACAGGCATACTAATAAACACTGCACTGTGTATTTAT
TCCAACGAGTGAATACACGGAGCAATGTCGCTCGTAACTAAACAGGAGCC
GACTTGTTCTGATTATTGGAAATCTTCTTTGCCCTCCAGTGTGAGGGCGATT
TTTTATCTATGAGGATATGAATAGATGTCAAACATCAAAAAATACATCATTGA
TTACGACTGGAAAGCATCAATAGAAATTGAAATCGATCATGACGTAATGAC
AGAGGAAAAACTTCACCAGATTAATAATTTCTGGTCAGACTCTGAATACCG
ACTCAATAAACACGGCTCTGTATTAAATGCTGTATTAATCATGCTGGCGCAA
CATGCTCTGCTTATAGCAATTTCAAGCGACTTAAATGCATATGGTGTTGTGT
GTGAGTTCGACTGGAATGATGGAAATGGTCAGGAAGGATGGCCTCCAATG
GATGGTAGCGAAGGAATAAGAATTACCGATATCGATACATCAGGAATATTTG
ATCCAGATGATATGACTATCAAAGCCGCCTGAGCGCGGCGTTACCGCATACC
AATAACGCTTCACTCGAGGCGTTTTTTCGTTATGTATAAATAAGGAGCACAC
CATGCAATATGCCATTGCAGGGTGGCCTGTTGCTGGCTGCCCTTCCGAATCT
TTACTTGAACGAATCACCCGTAAATTACGTGACGGATGGAAACGCCTTATC
GACATACTTAATCAGCCAGGAGTCCCAAAAAATGGATCAAACACTTATGGC
TATCCAGACTAAATTCACTATCGCCACTTTTATTGGCGATGAAAAGATGTTT
CGTGAGGCCGTCGACGCTTATAAAAAATGGATATTAATACTGAAACTGAGAT
CAAGCAAAAGCATTCACTACCCCCCTTCCCTGTTTTCCTAATCAGCCCGGCA
TTTCGCGGACGATATTTTCACAGCTATTTCAGGAGTTCAGCCATGAACGCTT
ATTACATTCAGGATCGTCTTGAGGCTCAGAGCTGGGCGCGTCACTACCAGC
AGCTCGCCCGTGAAGAGAAAGAGGCAGAACTGGCAGACGACATGGAAAA
AAGCCTGCCCCAGCACCTGTTTGAATCGCTATGCATCGATCATTTGCAACGC
CACGGGGCCAGCAAAAAAGCCATTACCCGTGCGTTTGATGACGATGTTGA
GTTTCAGGAGCGCATGGCAGAACACATCCGGTACATGGTTGAAACCATTGC
TCACCACCAGGTTGATATTGATTCAGAGGTATAAAACGGATGAGTACAGCA
CTCGCAACGCTGGCTGGGAAGCTGGCTGAACGTGTCGGCATGGATTCTGTC
GACCCACAGGAACTGATCACCACTCTTCGCCAGACGGCATTTAAAGGTGAT
GCCAGCGATGCGCAGTTCATCGCATTGTTGATCGTCGCCAACCAGTACGGC
CTTAATCCGTGGACGAAAGAAATTTACGCCTTCCCTGACAAGCAGAACGGC
ATCGTTCCGGTGGTGGGCGTTGATGGCTGGTCCCGTATCATCAATGAAAAC
CAGCAGTTTGATGGCATGGACTTTGAGCAGGACAATGAATCATGTACATGC
CGGATTTACCGCAAGGACCGTAATCATCCGATCTGCGTTACCGAGTGGATG
GATGAATGCCGCCGCGAACCATTCAAAACCCGCGAAGGCAGAGAAATCAC
GGGGCCGTGGCAGTCGCATCCCAAACGGATGTTACGGCATAAAGCCATGAT
TCAGTGTGCCCGTCTCGCCTTCGGATTTGCTGGTATCTATGACAAGGATGAA
GCCGAGCGCATTGTCGAAAATACCGCATACACTGCAGAACGTCAGCCGGA
ACGCGACATCACTCCGGTTAACGATGAAACCATGCAGGAGATTAACACTCT
GCTGATCGCCCTGGATAAAACATGGGATGACGACTTATTGCCGCTCTGTTCC
CAGATATTTCGCCGCGACATTCGCGCATCGTCAGAACTGACACAGGCCGAA
GCAGTGAAAGCTCTTGGATTCCTGAAACAGAAAGCCACTGAGCAGAAGGT

GGCAGCATGACACCGGACATTATCCTGCAGCGTACCGGGATCGACGTGAGA
GCTGTCGAACAGGGGGATGATGCATGGCACAAATTACGGCTCGGCGTCATC
ACCGCTTCAGAAGTTCACAACGTGATAGCAAAGCCCCGCTCAGGAAAGAA
GTGGCCTGACATGAAAATGTCCTACTTCCACACCCTGCTGGCTGAGGTTTG
CACCGGTGTGGCTCCGGAAGTTAATGCTAAGGCGCTGGCCTGGGGAAAAC
AGTACGAGAACGACGCCAGAACCCTGTTTGAATTCACTTCCGGCGTGAATA
TTACTGAATCCCCGATCATCTATCGCGACGAAAATATGCGCACCGCCTGCTC
TCCCGATGGTTTATGCAGTGACGGCAACGGCCTTGAACTGAAATGCCCGTT
TACCTCCCGGGATTTCATGAAATTCCGGCTCGGTGGTTTCGAGGCAATAAA
ATCGGCTTACATGGCCCAGGTGCAGTACAGCATGTGGGTGACGCGAAAAG
ATGCCTGGTACTTTGCCAACTATGACCCGCGCATGAAGCGTGAAGGCCTGC
ATTATGTCGTGATTGAGCGGAATGAAAAGTACATGGCGAGTTTTGACGAGA
TGGTGCCGGAGTTCATCGAAAAAATGGACGAGGCACTGGCTGAAATTGGT
TTTGTATTTGGGGAGCAATGGCGATGACGCATCCTCACGATAATATCCGGGT
AGGCGCGATCACTTTCGTCTACTCCGTTACAAAGCGAGGCTGGGTATTTCC
CGGCCTTTCTGTTATCCAAAATCCACTGAAAGCCCAGCGGCTGGCTGAGGA
GATAAATAATAAACGAGGGGCTGTATGCACAAAGCATCTCCCGTTGAGTTA
AGAACGAGTATCGAGATGGCACATAGCCTCGCTCAAATTGGAGTCAGGTTT
GTGCCAATACCAGTAGAAACAGACGAAGAATTTCATACGTTAGCCGCATCC
CTTTCACAAAAGCTGGAAATGATGGTGGCGAAAGCAGAAGCAGATGAGAG
AGACCAGGTATGACAACCACGGAATGCATTTTTCTGGCAGCGGGCTTCATA
TTCTGTGTGCTTATGCTTGCCGACATGGGACTTGTTCAATGACACCTCAGCA
GGAAAACGCCCTTCGCAGCATTGCCCGTCAGGCTAATTCTGAAATCAAAAA
AGCCAGACAGCAGTTTCCGGATAAAAACGTCGATGACATTTGCCGTAGCGT
ACTGAAGAAGCACCGCGAAACGGTAACGCTGATGGGATTCACACCGACTC
ATTTAAGCCTGGCAATCGGCATGTTAAACGGCGTCTTTAAGGAACGATGAA
CATGAAAAGCAAAATCATCAGGGAGCTACAGGCTCCTTTTTTTATTATTCGC
ATTCACCCTCAAGCGTATTAACCAACAATTCAGGGATTAATGGAAGATGGC
AGACATCATTGATTCAGCATCAGAAATTGAAGAATTACAGCGCAACACAGC
AATAAAAATGCGCCGCCTGAACCACCAGGCTATATCTGCCACTCATTGTTGT
GAGTGTGGCGATCCGATAGATGAACGAAGACGCCTGGTCGTTCAGGGTTGT
CGGACTTGTGCAAGTTGCCAGGAGGATCTGGAGCTTATCAGTAAACAGAG
AGGTTCGAAGTGAGCGAAATTAACTAGAAGCCAAAGATAAAATCATCGCTG
AGCAGGAGAAAATCGCTAACGGAGAAAAGACAGTAAGTCAGTATATGAAA
ACCGCATGATATCATCAGATAAAAATCGGTCGTAAAGCGAAATATTAATACC
AGAACAAACGAGTCGAGGTAAATTATATTACCTCGATAAATTAACTAAAACT
TGCCCGCTATATACTATATCATTCAGTATCATCACGCGCGGTCTGTGCATATG
TCACTACCGCACCTAATATATTAATTTTCTTTTCAACATAGATAATATTATCGT
ACTCATAATTGCCATACGGATAGCAAATGCGAATATTCTCATGTAGATCGGG
GTCATCCACCTCAGCTCCAGAACAACTTTTTGAACTACCGGAAGTATACCG
ATACGGTGCAACATAAGACGATGTCTCTCCAGGCAAAAAATAAGTTAGTGT
CGTAAGGAGTATAATCAGAAAAAATCCAGCAAATATGCACATCCCTGCATA
AACCTTAAGGTATGCTGACAGACTCTTCCAGCCTCTTTGTTTTACTATCCCC
TTCTTAACCCAAAACAGAGATAACAGAAAAGCTATTCCCATGCTAAACAGA

ATGTAATAGTGGGATATACTCTGATTAAGAAACGTGACCCTGTAGATATCTG
CCCGCCACCAGAAGAAAAGGAAAATAAAGATCAGCCCTGAAACTGTCATG
CAAATCAAATAAGGATACGAATCTTTTTTCATGTTTAGCGCCCATAAAATTTT
TCCTGACCCGGACAAATTTACCATCCATTTTTTGCGCAGAAAATAGCTCATT
ACTTACTGCACAATAATACACAAAATTGCGTAAATTTTTTGCATGGATTTTA
GCTCTTTCAGCCGACATTTAAGGGGTAAAGAGCATTTCCTAAAAGCAACTG
ACCAACCCAACAGAATGGGCTACCGCTTACGTTGAGAGCAAAAAAGTGTA
TAGCAGCAATGAACAGCATCCTCGCACTGACGAGGATTTCTTTTATCTGAA
CTCGCTACGGCGGGTTTTGTTTTATGGAGACAAGAAATGTCAGATTTGGCTA
TGAAGGTTTTGAAATGGCAATCAACTGGCGATGTCGGCATCAGTAGCGCAA
CTCTTGCCTCAATCGCATGTGGCCTGAAAAAGAATATCTATGGTCATCACTT
CGGCGCTCCCCATGACGCAGCAGACTTTCGGCGATGCGTTGCACTTGTTGA
GCAGATTCCAGAAATCAGAGATTCATTCGACAAGGTTGCAAAGCGCGTTCC
GGCATTCAAAGGAATCCTCAACGAATGGGATTCACTCGTTGCTCTGTTGAA
GTCTGAAATGAAGACGTACGGGAACAAAGCACCAGAGACTTACAGAAGAA
TCAGCGAGCTACGCAAGGACTAACCCGCCTCACACTCGATGAGGCCTGTTC
ATTTCTCAAGATATCCAGACCTACCATTGCTGCATCAATGCGGCTTTTTATTG
CCTGATTTGCAGGTTCGATTCCATATTCGGAGATAGCACTCATGCAACACGA
ACTACAACCTGATTCACTGGTTGATTTGAAATTCATCATGGCTGATACTGGC
TTCGGTAAGACCTTCATCTATGACCGGATTAAGTCCGGCGACCTGCCTAAA
GCCAAAGTTATCCACGGACGAGCAAGATGGTTATATCGTGACCATTGTGAA
TTCAAAAATAAGCTCTTAAGCCGCGCCAATGGGTAAAATAGCGGGTAAAAT
ATTTTTCACATCTAAAAAACACCATTCCAATCAATCCCCTGCCGCCTCAAGT
AGATGTCTGCAGGGGACACCATTTATCAGTTCGCTCCCATCCGTACCAGTCC
GCAAAATCCCCTGAATATCAAGCCTTCCGTAGATTCACAGTTCGTAATGGTT
CGCGTCAGATCGTTGACAGCCGCACTCCATGACGGGTAAAAAGTGGATAA
AATAATTTTACCCACCGGATTTTTACCCATGCTCACCGTTAAGCAGATTGAA
GCAGCAAAGCCGAAAGAAAAACCATACCGCCTACTCGATGGTAATGGCCT
GTACCTTTATGTCCCTGTGTCAGGGAAAAAGGTATGGCAGCTTCGCTACAA
GATTGACGGTAAGGAGAAAATCCTGACCGTAGGAAAATATCCGCTAATGAC
TTTGCAGGAGGCAAGAGATAAAGCATGGACCGCGAGGAAAGACATCTCGG
TTGGCATCGATCCGGTAAAAGCGAAAAAGGCTTCGTCTAACAACAATTCCT
TTAGTGCGATTTACAAGGAATGGTACGAGCACAAGAAGCAAGTCTGGTCA
GTAGGCTATGCAACTGAACTTGCAAAAATGTTTGATGACGACATTTTACCTA
TCATCGGCGGCCTTGAAATTCAGGATATTGAGCCGATGCAACTGCTGGAAG
TAATCCGCAGATTTGAAGATCGCGGTGCAATGGAGCGAGCCAACAAAGCC
CGCAGAAGATGCGGCGAGGTTTTCCGTTACGCTATTGTCACTGGTAGGGCT
AAATATAACCCGGCACCTGACCTTGCTGACGCCATGAAGGGATACCGCAAG
AAGAACTTCCCGTTTCTCCCAGCAGACCAGATCCCTGCATTCAACAAAGCA
CTGGCAACATTTTCAGGAAGCATCATATCGCTCATTGCGACCAAAGTTTTAC
GCTACACAGCCCTAAGAACGAAAGAGCTTCGCTCCATGCAATGGAAGAAC
GTCGATTTTGAAAACAGGATTATCACTATCGACGCCAGTGTGATGAAGGGC
CGCAAGATTCATGTTGTTCCTATGTCAGACCAGGTGGTTGAACTTCTCACTA
CGCTAAGCTCCATCACTAAACCAGTATCAGAGTTTGTTTTTGCCGGGCGCA

ACGATAAGAAGAAGCCAATCTGCGAGAACGCGGTGCTACTTGTGATCAAA
CAAATCGGCTATGAGGGTCTGGAAAGCGGTCACGGATTCAGGCATGAATTC
AGCACGATTATGAACGAGCACGAATGGCCTGCTGATGCTATTGAAGTGCAA
CTGGCACATGCCAACGGCGGATCTGTGCGCGGGATTTACAACCATGCTCAG
TATCTCGATAAGCGCAGAGAAATGATGCAATGGTGGGCGGACTGGCTTGAT
GGGAAGGTGGAGTAATCCGCCTTAACCACTATCGAAGAGCACAAAGCCTT
GCAATCCAGTACAAAGCTATGTGTGTCTCAGTTTTGTCCCTCTTTTTTGTAC
TAAAAAACATAGCAATTGAGGATAAACCTCATGCTATTTTCGCTTATATGCCT
TTAAAGGCATGGCGCTTAAATAGATAAAAACACCACAAAAGCATAAAAAAC
CACACAGTAAAACCGCAACATAAAACAATAACAGATAATTAAACCAAAAA
CAGATAGCGCATTGTGATAATCATTCAATACTAAACAAAATATAAACAGTGG
AGCAATATGTGATTGACTCATTAAGTTAGATATAAAAAATACATATTCAATCA
TTAAAACGATTGAATGGAGAACTTTTATGCGGGCGAAACTTCTGGGAATAG
TTCTGACAACCCCTATTGCGATCAGCTCTTTTGCTTCTACCGAGACTATATC
GTTTACTCCTGACAACATAAATGCGGACATTAGTCTTGGAACTCTGAGCGG
AAAAACAAAAGAGCGCGTTTATCTAGCTGAAGAAGGAGGCCGAAAAGTC
AGTCAACTCGACTGGAAATTCAATAACGCTGCAATTATTAAAGGTGCAATT
AATTGGGATTTGATGCCCCAGATATCTATTGGGGCTGCGGGCTGGACTACTC
TCGGCAGCCGAGGTGGCAATATGGTCGATCAGGACTGGATGGATTCCAGTA
ACCCCGGAACCTGGACAGATGAAAGTAGGCACCCTGATACACAACTCAATT
ATGCCAACGAATTTGATCTGAATATCAAAGGCTGGCTCCTCAACGAACCCA
ATTACCGCCTGGGACTCATGGCCGGATATCAGGAAAGCCGTTATAGCTTTAC
AGCCAGAGGTGGTTCCTATATCTACAGTTCTGAGGAGGGATTCAGAGATGA
TATCGGCTCCTTCCCGAATGGAGAAAGAGCAATCGGCTACAAACAACGTTT
TAAAATGCCCTACATTGGCTTGACTGGAAGTTATCGTTATGAAGATTTTGAG
CTAGGTGGCACATTTAAATACAGCGGCTGGGTGGAAGCATCTGATAACGAT
GAGCACTATGACCCAGGAAAAAGAATCACTTATCGCAGTAAAGTCAAAGA
CCAAAATTACTATTCTGTTGCAGTCAATGCAGGTTATTACGTAACACCTAAC
GCAAAGGTTTATGTTGAAGGCGCATGGAATCGGGTTACGAATAAAAAAGGT
AATACTTCACTTTATGATCACAATGATAACACTTCAGACTACAGCAAAAATG
GTGCAGGCATAGAAAACTATAACTTCATCACTACTGCTGGTCTTAAGTACAC
CTTTTAACGAAGTTAACCAGATTTTCTCCCCGGTGGTATTGTATAACCCCGG
GGAATTTGTTTACAGTTACAAGTAGGCAATATCCACTAATTTTGTAAGCATTT
AACCTATGTCATAACGTGTAATCTCTTACCGCAACACAAGACCTCAATATAT
TCAAATCTTATCTTTATTCCGATATTCAGAGACACAATGCGAAAAATAATTAC
TCATTTCAAAGTTGTTTTAACGTTACTTCTACCAGTAACCGTATCTGCCCAG
CAGATACAGTGGCAATCCTGTATGGCCAGTCAATTCAACCACTGGTTTGGT
GAGGAAAAACCGTCTCCTGACTTACTATGTGGTTATTTGTCTGTTCCATTAA
AATATACAGACACAGGCGGAGATGCTTCTTATGAAAAAAAATCACAAGTCA
AACTAGCGTTGACAAAATTGCCGGCAAAAAGCAAGCATAAAGGAAGTATC
CTGATAATAAGTGGTGGTCCCGGGTTACCAGGCATAAATCCTTATATTAACTT
TGACTGGCCAGTCACAAATCTTCGTGAGTCATGGGATATTATTGGATTTGAT
CCTCGAGGCGTCGGACAGTCCACTCCGACAATAAACTGCCGGCAATCAGAT
ACAGAGACTCAGGAAAACATAACCGAAAAGCAACAAGTATTAAATAAAAT

TAATGCCTGTATCCATAATACCGGAGCCGAAGTCATTCGCCATATCGGCTCT
AACGAGGCTGTATACGATATTGATCGTATTAGGCAAGCCTTGGGGGATAAAC
AACTGACAGCCGTGGCGTATTCGTATGGAACTCAAATTGCAGCCTTATATGC
AGAACGTTTTCCCTACAACGTAAGATCTATCGTTCTTGATGGAGTCGTCGAT
ATCGATGACCTGGAGGACAACTTCACATGGCAACTCAAACAGGCACAGAG
TTATCAGGAAACGTTTGATCGCTTTGCATCCTGGTGTGCGCGTACAAAAAG
TTGCCCGCTTTCTTCAGACAGAGATAAGGCAATAACTCAGTTCCATGAGCTA
TTATCAAAATTACATCACAAACCTTTATTAGACAGTAAGGGAGAAAATATAT
CTTCAGATGAACTCATATCATTAACAACAGACCTTCTGCTATGGCGTTCATC
ATGGCCAACCCTTGCAACTGCCATACGCCAGTTCTCTCAGGGGATTGTCAG
TAATGAAATTGAAACTGCGCTCAGTGCTCCGATAGCCTCAGAAGAGTCAAG
CGATGCTTCGGGGGTAATCCTCTGTGTAGATCAGGGGGATGAGCAATTAAC
ACCAGAAGAGCGAAAATCCCGAAAAGACGCTCTTGCGAATGCCTTCCCGG
CTATTAACTTTGACAATGGACGTTCCGATTCACCTGATTTTTGTGAATTATGG
CCAATACATAGCGACCTGAACAAAACTCGCCTGAAAAATACTGTTCTGCCC
TCTGGTTTACTGTTTGTAGCACACAAATACGACCCAACAACGCCCTGGATT
AATGCCCGTAAGATGGCAGAGAAATTTTCCAGCCCGTTACTAACAATAAAT
GGTGATGGGCATACATTAGCTCTCACCGGAGTTAATTTATGTGTAGATAAAG
CAGTTGTACATCACCTGATCACTCCACAAAAAATAGAAAATATATACTGCCC
AGGAAATTCTGAAGCAGAAATACAATAATTTCAAAATATTCCGCTATTTGCT
CCCAATGCAAAACATATATTGCATTGGGAGCATATTCATATTTTTGTTATTCTT
GTATGAAATCGTCTCATGTACACGGTCCTTTTTTCGCCGAAAACTGATATTA
CTTTAATCAAAAGTTGACGTGCCAGATTCGAAGTAAATTTATAAAATATCAA
ATCAATATACATTTTGCACCTTCGAGATAGTAATGGCTGTTACTGTAACATGG
GTTACAAATAGCTTCATACCCAGAGCTACTGATGTATCAACACGATTCAACA
ATACCCGATATTTATTCCATGCCTCCAGCAACGATCTTTCTTCCTCCGTTGCG
ATTTCCAGATCTACAGCATCCTGCAGTGGCGCAATATACTCACTGAATTCCT
GGATGTAGAACTGTGTGGTGACGGTCTTCCAGCCATTCGGCTCCTGCTGTA
TCGAAGCATACCAGGCTATTTCAATATCGCTATGCTGCGGCAGCATTTAACC
CCTTGTAATTCATCGCCATAATTGATTTAATTCACAAATAAAACTATAACATG
GTGAAATTAATGAAAAAAAACACAGATGATGGGGCTAAAATTTACACACCA
CTTACCCTAAAGCTTTATGACTGGTGGGTTTTGGGAGTATCAAATCGGCTTG
CATGGGGATGTCCTACAAAGGAACACCTTCTTCCACACTTTCTGGAACATT
TAGGTAACAACCATCTGGATATTGGTGTTGGAACTGGGTTTTACCTTACTCA
CGTACCTGAGAGTAGTCTGATATCTTTAATGGATTTGAACGAAGCTAGCCTG
AACGCGGCATCTACAAGGGCTGGGGAATCAAAAATTAAACATAAAATTAGC
CATGATGTTTTTGAACCTTATCCCGCGGCGTTACATGGTCAATTTGATTCCAT
TTCCATGTTTTACCTTCTTCACTGCCTGCCTGGAAATATATCTACAAAAAGC
TGTGTAATACGCAATGCGGCGCAGGCCTTAACTGACGATGGAACTCTATAC
GGAGCCACAATTCTTGGCGATGGAGTTGTGCACAATAGCTTCGGTCAAAAA
CTGATGCGCATTTACAATCAGAAAGGCATCTTTTCAAACACAAAAGATTCC
GAAGAAGGCTTAACACATATACTCTCAGAGCATTTCGAGAATGTTAAAACC
AAGGTTCAAGGTACTGTAGTAATGTTTTCCGCTTCAGGGAAAAAATAGCAT
CCAACCGCAGCACGTTCTTGCTTAAGACGTGCTGCGGCATAATCCCAATGA

TTACTCCCTGACAGGGTTCGTAGGCCACTCAATATCAGGTGCAGTTGATGTA
TCAACACGGTTCAGCAACACCCGATACTTTTTCCAGGCTTCCAGCAACGAG
GTTTCTCCCTCCGTTGCGATTTCCAGATCTGCAGCATCCTGAAGTGGCGCAA
TATGCTCACTGGCTACCTGCATCAGGCTGTTTTTTGTTTCTTCCGCCTCCCG
GATCCGGAACAGTTTTTCTGCTTCTGCATCTTTCACCCAGGTTGTGCCGTTC
CACTTCTGAAACTCCCCTTCCGGGGATAACCAGGTGACATTTTCCGGTAAT
GAGCCGAGTTCAGAAATAAATAACGCGTCGCCGGAAGCCACGTCATAGAC
GGTTTTACCCCGATGGTCTTCAACGAGATGCCACGATGCCTCATCACTGTTG
AAAACAGCCACAAAGCCAGCCGGAATATCTGGCGGTGCAATATCGGTACTG
TTTGCTGGCAGACCTGTATGAGGCGGAATATATGCGTCACCTTCACCAATAA
ATTCATTAGTTCCGGCCAGCAGATTATAAATTTTTATGGTCCGTGGTTGTTCA
CTCATTCTGAATGCCATTATGCAAGCCTCACAATATAGTTAAATGCGATGTTT
TTGACGGTGTTTTCCGCGTTACCAGCAGCGTTAACGGTGATGGTGTGTCCA
TGTGAGCCAATCGCAACCGAGTGCGTATGAGCACCAATACCGACAGTATGT
GCGTGTGCACCTGCGCTTGCAGCAGTGCCGGACAGTGAGTGGGTATGTGC
GCCAGCAGATGATGTTGCATAGTTTTGATTATGCACAACAGACAATCTTGTT
GATGCACTACCAGCACCGGAGTTAGCACTAGCCGTGTTCACGTTGGCTAGT
GAGTGTGTGTGTGCTCCAGCCGAGTTTGTAGAGCCGCTCACACTGTGTGTA
TGTGCCCCGGTGTTATTCGTAGATTTAGTGCCGTAATCAAACGACGATGTGG
TTTTCGTACCCAAATCCGTACTTGATGCGCTGGCGCTGTGGGTGTGCGATTT
AATGCCGTCCTGTTCCTGAGACAATACGGCACGACCACTGGCGGGCTTGCC
CTTAATCGTCCAGCCACGCATATCAGGGATCACGCCTGACGGATAAGCAAC
TGCAAGTTTCGGGTATGCAGATTTGTCAAAAGTCTGCCCCTGCATCAGGGC
ATAACCAGACGGAACGGTATCTGATGGCCACGGAATTGGTGCGCCAACTGG
GTAGCTTTCTGGTGGAAGATTTTTCGAGGTATAAACTTCTGCCCAGTCTTCC
TCAAAACCATAACCGTCTCTTGAAGAACGGTAGAACAGACCACCATTTCTG
TAATGCGCCTTCATCTGCAGGGTCCGGCAACTTCCGACTCCGGTATAGAAG
TTAACCAGAATATAGCTGTCGCCAGAGCGGGTGACATTGTAAGCGCCTGAT
TCGGCATTCCATGGAACGCCACCATCCGCATCGGCATATGTATCCGTTGCCC
TTCTGGCAAAAGCAGCCACATGCGCGGCGGTTAAAGTAATATCTTTGGAAC
CATCAAACTCAACACCAGAAACCCGTCTTGGCGTTTGCAGCTTTGTTGCTG
TTAATGCATTACCGTTCAGACTTGCGGACAGTTTGGTTCCAATAACCAGTTC
GCCGGTTGCGTTATCAATAGCAAACGGTCTTAATGTATTCCAGCCACCATAA
ACATCACCTTGATTGGTAAGCAGCAGGTAAGTTTTAGCGCCATCATTACGCC
ATAATGCACCATACTCCCCACCTATCATTCGAATCTGATTACCACCACGCGCT
ACAATTTCGTCTGTGGCAAAAAGTTTTTTGCACGACAAGTTATCGTTAACG
ATTAACGAATGAGACTCATAAAAACCACGCCCACTCTTAAAATCAAGGATA
ACGTCCGCCGCGATACATTCAGTCGCCGGATTTGTTGCCCCAAACTTATAGG
TCGTATCATTAACAACGAGATCAGCACCAGGTGCGGATATTGACAGGCCAT
CTTCAATAAACGCAAAAACAGGGAAAGCAGCGCCATCAACATAGAACACA
GAGCGCAAATCATCGCCCTTATTACTCATCATTATTGAGTGAATGGCTCGTT
CATTGTTTTGATATTGCCAGAACATGCCATAAGCATAACGCCCCCTGTCAGT
CCAGCCACCAGGCATAACAAATCCGTTAAACTCGCAGTTATTCATCGGATC
GCCTGCGGTTCGCGTTGCCGTGGTGATAATGACTCTTGATGCCAGTTCGCTT

ACTGAGCCAGCAGAACGCATAACAACAACAGGGTAATATTTTCCAGATGTT
GCACCTGCAGGAGCGTTAACCCGCACATAACGCATACCACGCTTATCAGCA
AAGTCTGTTTTACTGACCGCGTTAATGTTGTTCAGGAAGCATCCCTTATCGG
GTATATCAGCGCCGTTCTGGTCTTTCTGCAGACGTTTCTCTGCATTGTCATA
GGCTGATTTTACTGCCTTTGGCGTTGCCGCCAGCGTTTCAGACGTACTGTTG
GTCGCACTGCTGAGCTGTACTATCCCCTTTTTCGTCGTACTTGCATCCTCAA
GCGCCACGGCGGATGCAATATCCTCTGCCCGTTTAGCTGCTGTCTCGGCGC
GCGTTGCCGCGGATTCCGCCGTACTTTTGCTCTGAGCTGCCGCCGTCGCAC
TGCCAGCAGCCTCTGTCGCCTTCGTGGATGCCGTCGTGGCGCTGCTCTTCG
CTGCTGACGCTTGTCTGGTCGCCTCATCTTTTGAAGCAGACGCCGATGATG
CCGATGACGCCGCCGAACTGGCTGACGATGCGGCAGCCGTTTTTGAGGATT
CTGCGCTTGTTTCCGACGCTTTCGCGTTCGTTTCGGATGTCTTCGCTGCGGA
AGCAGACCTCGCTGCTGCGCTGGCCTGTTCAGTGGCTTCGCCAGCCTTCGT
TGTGGCTGTTGAAGCAGACGATGCGGCGCTTTCTGCCGATTTTCCGGCGGC
GGTGGCACTGGCTGAGGCCTGCCCGGCACTTGTTGACGCTGCACTGGCAG
ACGACGCAGCCGCTGTTTTTGAGCCTGCCGCAGCCGAGGCGCTCTGTCCC
GCTGCCGTTTCAGAAGACCTGGCGTTCGTCTCGGACGTTTTTGCCGCCTTC
GCGGAATTTCCTGCCGCCGTTGCCGAGGAAGCTGCACTACTGGCGCTTGAT
GATGCGTTCGTTTCTGATGATTTTGCCGCTTCTTTTGAGGCCGCCGCATCCC
GGGCCGAGGTCGCAGCTTCTGATGCCTTCGTGGTCGCGGTGGATGCAGATG
TGGCTGCTGATTGTTGTGACGCTGAAGCATTCGTTTCTGACGTTTTCGCCGC
ACCGGCACTGGTGGCCGCCGCGCTTTTTGAGGACTCTGCAGCGGCAGCAC
TTTTTGATGCTTCAGTGGCCTTTGTTGATGCCGTTCCTGCGCTGGAAGACGC
TGACTGAGCCGACGACGCGGCCTGTCCGGCTGACGTGCTGGCTGCGCGTG
CTGAGCCTGCAGCATCAGTCGCATGGGTTGCCGCCTCACGGGCTGATGTGC
CGGCATCGCTGGCTGACTTCTTCGCGGCTGCCGTGTTCTGTGCCACCGCGG
ACGCGTTACGCGCCACCTCTTCCACCATCAGTTCAAAACGGCGCAGTGCCT
CAGGACGGGCATCATCCTCCGTCATGGCACCGAGAAAATCATTCAGCGTAC
CGGGTCGGGAATCTTCATACACGGTGATGGTCCCGGCATGTGACGGCGGGA
ATCCCTCCACCAACAGAATAACGCTGTACTGCCCGTACTCAACGTCCATGC
TGTAACGCCCGGCTTCATCCGGATTTTCTGAGGCCAGCGTGTTCACCACCA
CCGTGGTGCTGTTACGTTTTGCTTTCAGCTGGATTGTGCAGTTCTGTACCGG
TTTTCCTGTGCCGTCTTTCAGTACACCTGAAATCTTTACTGCCATATTCACCC
CACAAAAAAGCCCGCCTGAACCGGCGGGCTGTCATAACACTGTGTTACCT
GGCTAATCAGAATTTATAACCGACACCCACGATGAAACCGTCAGTGCGCCA
GTCACCACTGCCGGAGCCTTCATAAGCAATATCAATGGCCACGGATTCGGT
CGGGTTAAACTGCACGCCAGCCCCCCACGCCAGAGACGTGTTGCTGTGGC
GACCGTCATCACTTCCGGTCAGCACATCGTGCTTTTTCCCCTTGTTGTCAGT
TACGCGAAGATAATCCCCGGAGAAAGTCGACACACGGCTGTAAGCTACAC
CCGCCATCGCATACGCGCTGAACCATTCATTCACGCGCACAGACGGCCCCG
CCATTACGCTGAACCAGCGGTTACGAACGGAATCTTCATGCCAGCGGGTAT
CGCTGTAACGGGTCAGCTGGCGATTCTTGTCTCCTGCATAGCTGAATGACG
TCACCAGCCCCAGTGTGTCCGTAAACTCATAACGGTATTTCACGTTAATCCC
GTTCAGATCATCACTGCCGGGGACGTTCGTCGAGGCATGAAGATACCCCGC

GCTCAGCGTGGACTGATGTTCAGACGCCCATGCAGGCGCACCGGATACGG
CCAGACAAATGGCTGCGGACAAAATTGCTGCACAAACTTTACGCATAATTA
CCTCTCGCTTTTCTGCAATAAAAAAGGCGTCATTCCTGACGCCCTTTATTGG
GGTTATAAATATTTCAACGAATACTGATGCCGGAAGCGGCTTTTTTGGTCAC
AATCACCGTACAGTCGGTGATATTGCCTGCCCCCTGATTGCCTTTCTGGAAA
ATCTTAAACTCCAGAGTGACGCTTCCCCTGCCACTCGGCATATCAATAACTG
CACTGTAACTACCGGGAATGGCCCCTTTAGTTTCTCTGGATGCGATTAATAC
GCCGTTTTTGCGAACTTCAAAACCATAACCCGTGTATCGCGTGCCTCCTGG
GTTATTTCCGCTCCCCGGATCGTCATACGCTATACCGTTAAAAATAATGGGC
GGAATAATAATCTGGCGGTCAAAGTTATGATCATCGCTGATGGTGACTGTAA
CCGTACCGTTTGGTGTTTCCGTGTTACCCCACGTACCGACTTTTTTCGGGAA
GGCTTTTGATACAGCTTTAACGAAATCTCCTCTGACCTGGGTCGCCTCCAG
CATGCCCTTAATCGTACAGTTCTGGTTAATCGTGACATTGTTGAGCGTTCCT
GAGTTCGCATTCACACTGCCACTGATATCCGCATTTTTTCGCCGTCAGTCTC
CCGTCTGATGTCAGGGAAAATGCCGGAGGATTACCGCCGCTGGTAATAGTG
GGGGCCGTCAGGCGTTTCAGGAACACTTCATTCATGAATATCTGATCGCCCT
GACCAACAAACATCGGCTTTGTGTTGCCATTCGCAGGATTAATCATCGCAAT
CCTGTCTGCTGCCAGCAGCACCTGACTCTGCATGCCGTCAGGGGTGTTCTC
AATACCGGCACCGATACCCGCAATATAAAGGCGTCCGTCCTGCATCTGCTGC
AGCTTCACTGCCCACATGCTGTTCAGGTTATTATTTGTATCAACCTGAACCT
TCTGTATCTGCTGGATCGCTGCACTCTGGTCTTCCAGTTTCTTATTGACGGT
CTGTGTTATTTCATTGCTGACATCCGTTATGGACGTCCTGATTTCAGTCAGG
TCAGGCGCAAGCTGACCGTTATCAATCTGCGTCCACAGCTCCTGAGCCAGA
TGGGTTTTCCCTATCTCGCCTTTGAAAAAATCCAGATAGCCTGATGCATCAT
CACTCGGCTGACCGACAGCCTCCACGAATGCCGATTTGCCAACGGTGTTCA
CACTGCGAACGTAAAAATAATAATCATGGCCCGGTTTGATATTGATACTGGC
GGCTATCCAGTACAGTGCCGTACCAAGATAACGCGCGCTGGTTTCAACCTG
CCTGATATCCGCAATCCGTTTTTCCGAGAACCAGAACTCAAACTGTACCGT
CGGGTCATAAACCGCAAGATGCGGCGTGGCGGTTATCTGAAAATAGCCCGG
CGTCAGCTCAATCCGCGACGGCGCTGCCGGTGCGGCAATCCGGAACGATA
CCGACGCCGGATCGCCCTGCTGCCCCCACGCATTTACTGCCCGGACTGTCA
GCCTGTAGTTCCCCAGAGCCAGTTGTGTGAAGCGGTAAGTGGTTTCCGTCG
TCCGGGCCGTGCTGACCAGCCGCTCACGGCCGTCATCCGCTGCCACGGTC
AGGCGAAGCAGGAAGCTCACGCCCTTCACCACCTTCGGCGTGTCCCAGCG
GGCCAGCACCTGGTATTCCCCGCTGTCTGCGGTGACTTCTGCGGTCAGGTG
CTGCACCGCTGGCGGCGTGACACCATTTACCGTGCCGCTCTGGTCGCCGTC
AAAGTGCGCCCCGTTATCCACGATGGCCTCTTTTTCCGGTACATGCTGCACG
GCGGTGATGGCATACGTGCCGTCGTCGTTCTCACGGATACTCACGCAGCGG
AACAGGCGCTGGCGCAACGTCGGCAGCTTCAGCCCCCATACGCTGTATTCA
GCAACGCCGTCAGGAACACGGCTCACTTTCACCTTCACGCCGTCGGTGAC
GGACTGAACCTCCACGCTGACCGGACTCCCCTGCCCGTCAACCAGGCTTAT
CAGCGTGGTGCCGGAAGATGGCAGCGTGATTTCACGGTCGAGCGTCAGCG
TCCGGGTCTGGCTGTTTACCGCCAGCACGCGCCCGCCGGTGCGGATACCGG
CATAGTCATCATCGCAGATTTCAATGACATCGCCCGGTACATGGCGAAGCCC

TTCGGCACCCACGCTGAAGTCCACGGTCTGCGTTTCCAGCAGTTCTGTTTT
AATCAGCCACAGCCCGGCGCGGTGTGCCTGCCCCCGGCTGGTACAGCCAA
AAGCATCCATCTTCGTGACGTTACGACCGTAACGGGCAATGGCCTGCGTGT
CCTCCACAAGCTCTGTCGCCGTCTCCCAGCCGTTATTCGGGTCAATCCAGTT
CACCTCAACGGCATTATGGCGGTCCTTCAGGGCGCTGAAGCTGTAGCGGAA
CGGCGCACCATCATCCGGCATCACCACATTACTGCGGTTATAGGTCCACACC
TTATCCGACGGTCGGTCCTGCACGAACGTCAGCGTCTGCCCGTTCCATACC
GGCATACAGCGCATCGCAGAGCAGAAATCACTGAGCACATCCCACGCCTTG
CGCTGTGTGGTCAGGTACGCATTACAGGTGATGCGCGGCTCCGTGCCGCCA
AAACCGTCCGGCACCGACTGATCGCAATGCTGGCCGATGACATACAGCGCC
CATTTGTCCACATCCGCCGCACCAAGACGTTTCCCCATGCCGTAGCGCGGA
TGGGTCAGCATATCCCACAGACACCAGGCCATGTTGTTGCTGTATGCTGGC
TTAAACGTTCCGTCCCAGATACCGCTGTATTGCCGCGTCTGCGGGTTATAGT
TCGACGGCACCTGCAGAATGCGCCCGCGCAGATGATAATTACGGCTCACCT
GCTGGCTGCCGAACTGCTCCGAATCCACCTGCACGCCGACCAGTGCCGTG
TTCGGGTAGCACTGTTTCACATCGATGATTTCGGTGTATGACGACCAGAGC
GTTTTGTTCTGCAGCTGGTCTGTGGTGCTGTCCGGCGTCATCCTGCGCATCC
GTATATTGAACGGGCGCGGCGGCAGGTTACCCACCACCACCGAGGCCAGAT
ACTGCGAGGTGGTTTTGCCCTTAATGGTGATGTCTTTTTCCGTCACCCAGCC
ACCATTACGCTGGATCTGAACCAGCAGGCGGACTTCCGACGGATTCCGGTC
CCCCTTTGAGGTGGTTTCCACCAGTGCCTGCACGCCGAAGGTAAAGCGCA
GACGGTCGATGTTTGCAGACGTAATGGTGCGGGTGATCGGCGTGTCATATT
TCACTTCCGTACCCAGCACCGTCTCGGAGCCGGAGGATTCAAACCCCTCCG
GCGGTGTCTGCTCCTGCTCACCGGCACGGAACACCACCGTGACACCGGAG
ATGTTGGTATTCCCCTCAGTGTCCAGCACCGGCGTACTGTTCAGCAGCACG
CTTTTTAATCCATCCACCGGACCTTCAACCGGCCCTTCGCTGATGGCATCGA
TCACACTCAGCAACTGCGTGGACTTCAGGTTGTCCTTCGCTTCGCGCGGGG
TATGCCCCTTACTGCTTCCTTTACCCATTCCTCACGCTCCATAAATGACAAA
ACCGCCCGCAGGCGGTTTCACATAAAACATTTTGCATCAGCGACCAATCAC
CACAACCTGACCACCGTCCCCTTCGTCTGCCGTGCTGATCTCCTGAGAAAC
CACGCGAGACCCCACGCGCATTTCCCCGTACAGAACAGGCAGAACATTGC
CCTGGGCAACCATGTTATCCAGTGAGGAGAAATAGGTGTTCTGCTTACCGT
TATCCGTTGTCTGTGTACGGGGAGTTCTGGCTTTCGGTGCCAGCATCTGCGC
CACACCACCGAGCACCATACTGGCACCGAGAGAAAACAGGATGCCGGTCA
TACCACCGGCCCCAATGGCTGCCCCCCATGCTGCAAGGGTGGCTCCGGCGG
TAAAGAATGATCCGGCAATGGCGGCAGCCCCCAGGACAATCTGGAATACGC
CACCTGACTTGGCCCCGGCGACTCTGGGAACAATATGAATCACAGCGCCAT
CAGGCAGAGTCTCATGTAACTGCGCCGTTAACCCGGACGTGCTGACGTCCC
GCCCGGCAATCCGTACCTGATACCAGCCGTCGCTCAGTTTCTGACGAAACG
CCGGGAGCTGTGTGGCCAGTGCGCGGATGGCTTCAGCCCCCGTTTTCACAC
GAAGGTCGATGCGGCGGCCAAATCGTTGCAAATCCCCGTAAAGGCAGATG
CGCGCCATGACCGGTGACGCCAGAGGGAGTGTGTGCGTCGCTGCCATTTG
TCGGTATACCTCTCTCGTTTGCTCAGTTGTTCAGGAATATGGTGCAGCAGCT
CGCCATCACCACAGTAAATGGCGGCATGATTCGGCACCGATGAACCAAAAC

AGCACAGCAGCACATCGCCCGGTTGTGCTGATGACAACGGCACCTGATAC
AGCCCTGTGGCCTCCAGATTATCCAGATAGAGATTCTGACCGTGACGCCAC
CAGTCATCCCCGCGATGAAAATCCGGCATCTCAATCCCCGCCAGATGATAA
GCATCCCGGAACAGCGTGTAACAGTCCGTCACCCCGTGCTCAAAGCGCCG
CCCGGTGAGATGCGGCACACAGCGGAACTTGTGAATCGCCCCCCGGCAGA
CCAGCCACCACGGCAAATCACTCTGCACCTGCAGCCGCCGGTCAGCCTCA
CTCAGCCAGGGCAGACCACCGGGGTGGCTGTGGACCAGCGCCACAATCTC
ACCCTGCATTTCTGCCTGCAGCCAGTCTTCCGGCGACATACGGAAATACGC
CTCCGGCTCACCGGAGATATTCACGCAGGGGAAATATCTTTCCCCCTCCGG
CGTGCTTACCACGAAGCCGCACGACTCCGCTGGCGCACATCGCCGGGCGT
GCGCCAGAATCGCTGATTCTGTCTGTGTCATGGGATTTACTGCGAAAGTTTG
TTAATGGAAAGGAAGCCGCCAAAGTTGCCGACGTTATTGCGAAACTTACAG
CCGCTCAGGCATTTGCTGCATTTATCCTTCGTGATATCGGACGTCGGCTGGT
CATATTCATCCGCGACTGCCGGACCGTGATAACCGCACTCATCACCGCGATA
GGTCCAGGTGCAGGTGTTGGCCAGCATGATACGTCCCGGAAAAACAGCGC
CATCCGTTTCCGTCGGCGTGGACAGTACAAAGGAGGCACTCACCGCGCTC
AGTTCGCTGCACTGCTCGATGCGCCAGCGGCTGATCACCTCCTGCTCCGGA
TCGGCGTCACTGTTTCCGTTGACGAAGTTCACCGCATCCAGAAAACGGGC
GTAAACCTTACGCCGGACCACCGTTCCGCCGACCAGACTCTGCAGATCTTC
CGCCATCCCGGTGACCATACCGTACAGGTTAGAAACCGTCAGCGTGGGGCG
CGTACTGGTGCCTTTGCCATTCAGTTCAAAACCACTCCCCTGAATGGGATA
CGGCTGATACTGTCGCCCCTGCCAGGTGACCGGCACACCTTTTTCGTTCTG
CTCATTACAGAAAAAATAACGTTCTCCACCGACCTCTGTCAGGTCGATTTC
CCAGAGCACCACGCTGGCCGACTGCTCCGCACGGGTGCATTCATTCAGTGT
TTCCTGCCGGATATCCTGCATCAGTTCACCACCTGTTCAAACTCTGCGCTGA
ACTCAACACGCAGCATACTGACCCGCGACGACCATTTTGCGCAGGTCACCT
TTATCTGCCGCCACTCATAAGGCGGCGTCCACAGAAAGGCTTTCCAGCCCC
CGTGCTCTTCCAGAAACGACTCCAGTACCGTGGCCTCCTCACGGGGGACA
GAAAGCGTCACGCTGTACGTTTTCAGGTTGGCATTCAGCCCGGCAGGCGCT
CGCTGGGAATAGCCATCACCAAAGCGCACCTTTCTTACAGAAGGGGCCGA
AGCCACATCCATACCGGGTTTCACTTTCCAGCGGAAGGTTTTCATCGTCCA
CCTCCGGAGAACAGTCCACCATCACGCATCTGTGTCTGAATTTCATCACGG
GCACCCTTGCGGGCCATGTCATACACCGCCTTCAGAGCAGCCGGACCTATC
TGCCCGTTCGTGCCGTCGTTGTTAATCACCACATGGTTATTCTGCTCAAACG
TCCCGGACGCCTGCGACCGGCTGTCAGCCAGACTGCCCGGTGTACCGACAT
AACCACCGGTGGCATAGCCGCGCATCAGCCGGTAGAGATTCCCCACGCCAA
TCCGGCTGGTTGCCTCCTTTGTGAAGACAAATTCACCACGGTGAACAATCC
CCGCTGGCTCATATTTGCCGCCGGTTCCCGTAAATCCTCCGGTTGCAAAATG
GAATTTCGCCGCAGCGGCCTGAATGGCTGTACCGCCTGACGCGGATGCGCC
GCCACCAACAGCCCCGCCAATAGCGCTGCCGATACTCCCGACAATCCCCAC
CATTGCCTGCTTAAGCAGAATTTCTGTCATCATGGACAGCACGGAACGGGT
GAAGCTGCGCCAGTTCTGCTCACTGCCGGTCAGCATCGCCGCCATATTCTG
TGCAATACCATCAAAGGTCTGCGTGGCTGTACTTTTTACCTGCGACATACTG
TCCGTGGCGCTCTCTTCCCACTCACTCCAGCCGGACTTCAGGCCTGCCATC

CAGCTCCCGCGAAGCTGGTCTTCAGCCGCCCAGGTCTTTTTCTGCTCTGAC
ATGACGTTATTCAGCGCCAGCGGATTATCGCCATACTGTTCCTTCAGGCGCT
GTTCCGTGGCTTCCCGTTCTGCCTGCCGGTCAGTCAGCCCCCGGCTTTTCG
CATCAATGGCGGCCCGTTTTGCCCGTTGCTGCTGTGCGAATTTATCCGCCTG
CTGCGCCAGCGCGTTCAGGCGCTCCTGATACGTAACCTTGTCGCCAAGTGC
AGCCAGCTGGAGTTTGTACTCCAGCGTCTCATCTTTATGCGCCAGCAGGGA
TTTCTCCTGTGCAGACAGCTGGCGACGTTGCGCCGCCTCCTCCAGTACCGC
GAACTGACTCTCCGCCTTCCACAAATCCCGGCGCTGCTGGCTGATTTTCTC
ATTTGCTCCGGCATGCTTCTCCAGCGTCCGGAGTTCAGCCTGAAGCGTCAG
CAGGGCAGCATGAGCACTGTCTTCCTGACGATCGCCCGCAGACACCTTCAC
GCCGGACTGTTTCGGCTTTTTCAGCGTCGCTTCATAGTCCTTTTTCGCCGCC
GCCATCAGCGTGTTGTAATCTGCCTGCAGAATTTTCCCGTCCTTCAGTGCCT
TGTTCAGTTCTTCCTGACGGGCGGTATATTTCTCCAGCGGCGTCTGCAGCCG
TTCGTAAGCTTTCTGCGCCTCTTCGGTATATTTCAGCCGTGACGCTTCAGTA
TCGCTCTGCTGCTGCGCATTTTTGTCCTGTTGACTCTGCTGTTCAGCCTTCT
TTCTCGCGGCTTCAAGCGCAAGACGGGCCTTTTCACGATCATCCCAGTAAC
GCGCCCGCGCTTCATCGTTAACAAAATAATCATCCTTGCGCAGACTCCAGAT
GTCGTCCGCTTTCTTAAACGCAGCCTCTGCCTTAATCAGCATCTCCTGAGCG
GTATCAGGACGACCAATATCCAGCACCGCATCCCACATGGATTTGAATGCCC
GCGCTGTCCTGTCTGCCCAGGTCTCCAGCGTACCCATGTTCTCTTTCAGGC
GGCGGGTCTGGTCATCAAACCCTTTCGTCGCGGCCTCGTTCGCCGCCTGCA
ATGCCCCGGCTTCATCGCCGGAACGCTGCAACTGAGCAACATACGCAATCT
GCTCCGCCGTCACGTTATGGAACTGGCGTGCCATCGCTGTCAGCCCTGACG
TCGGGTCTGTGGTCAGCTTCCCGAAGGCTTCAGCGACCTTGTCCACCTCCA
CGCCGGATGCAGAGGAGAAACGCGCCACACTCTGGCTGATGGATGCAATC
TGAGCCTCACCGCTTACTCCCGCCTTAACCAGTGCGCTGAGTGACTCGCTG
GTCTGGTTAAACGTCAGCCCTGCCGCCTGCCCGGCTCTGGACAGGACCAG
CATACGATCTGCCGTCAGACCCGACTGATTGCCGGAAAGGACCAGCGTTTT
GTTGAAATCGGACAGGGTTGAGTTGCCCTGATACCAGGCATACGCCAGCGC
ACCGGTCGCCACCGCCAGCGAGGTGGCCCCGACCATCGGCAGGGTGATCG
CACCGGCAAGCCCCCTGAACATGGGGATCATCCCGCCGAAGGAGTCCTTC
ACCTGACCACCCTGTTGCAGCAGGATCAGCCACGGACTTTGCCTGCCTGCA
AGCTGCGTGGCCACGTCGGTGAACTGCGCAGGCAGCATACGCATGGCAGC
TTTATACTGCCCGACGGAAATCCCCGCTTTCTGTGCAGCCAGCGCCTGTCG
GCTCAGCGACTGTTCAACGACTGCCGCTGTTTTTTTCGCATCACTTTCCGTA
CCGGAAAAATGACGCCTGACTCTGGCCATCTGCTCGTCAAATCTGGCCGCA
TCCAGACTTAAATCAACGACCAGATCGCCTACCGGTTCAGCCATACCGGAC
TCCTCCTGCGATCCCTTCTGATACTGTCATCAGCATTACGTCATCCTCCGTCA
TGTCCGCCACATCCGGGGAAACGGGGATAACTTCATTCACGTCCGGGCCAA
AGCGGACGCCTCCGGCAAGCCCTGCCGCTTTCTGCATCAGCACATCATCTT
CAGGCTCTTCGTCAGCCTCACGCCGGTTCAGCAGACTGAAATCCAGCGGAT
GCATATCCGGATCGCTGAAAAACAGGCTGAGCACGGTGTACGTCAGCCCG
GAAAAGTGCATATCCAGCAGAACATCATGAAAATAATGGGTACTGTAAAAG
CGGTGCCAGTCGGCATACTCCGTGGATGACATCCCGGCAAGCATGGCACGC

CAGTCGGGTCGCCCCATCTCGCGCGCCAGTTTCAGGGCAAAGCTCAGCTC
ACCGTCGAACACTTTCCCGCAGAAACAGGCTCTGCAGGCCCGGCGTCCTC
TGCCTGTTCAGGGGCATCATTCACCACAAACTCATACATACCGGACAGCCG
GTACACCACGTTTTCAGCATGAGAAATTGCCTCTGTGGGCCAGGTGGTAAG
CACTTCCTGCTCAATCTGTTTAACGGCTTCATTCATGGAAGGCTGCTTTGTC
TTCTGCGGATGGTTATGCCACAGGGACATAGCCACCACAAAAGCACCGGTT
CTGATGGCGTCTTCCACAGTAAACTTCCGGTTGCTGTCTGACTCCGCCTGTT
CTGCCTGCCGTTTCATCAGGGCGAGATGCTCAATACGCTGCAGGGCTGACA
GTTCAGAAAGCGTGACGGTCACGCCGTTATGTTCAAATGATTCGGTTTTCA
GGAACATCGCTGACTCTCCGGATTAACTGTCGGTGACAGTGATTTCTGCAA
CCGCAGCAAGTTCACCATTACCGGATACAACCGGAATGTTGACCTTACCTG
CAGCAACGCCTTTCACGGTGATGGTCATACCACTGACCGACACGGTGGCTT
TTGTTTTATCCGCAGACACCGCACGAAAGCTCTTGTCGGTTACGCCCTCCG
GCTGGAAGGCCACGGTCAGCGTGGTGCTCTGCCCTTTCACCACCGAGGTG
CTGGCAGGCGTCACGGTCATGCCGGTTGCCGCTGTTACCGTGCTGCGATCT
TCTGCCATCGACGGACGTCCCACATTGGTGACTTTCACCGTGCGGGTGATC
ACTTCCTTCGCCGTCACCGCCTTACCGATACTGCTGACCCAGCCACGGAAC
ACATCGACCGTGCCGTTCGGGAAGCGGATTTTATAGGCACGGGTATCGCCT
TCATTAAACCACGCCAGCAGCGCCTGCTGCCCCTGCTCTCCGGGCATCCAC
GCCAGCGTGAAGCTGGTATCTCCGGCAGATTTCTGCCCCTGCCCGGTCGCA
GTCCAGTCTGCATCTTCATCATCGAGATAGCTGTCGTCATAGGACTCAGCGG
TCAGTTCGCCGGGCGTCAGGTCTTTAACTTTTGCCAGACGCGACCAGTCAA
CGTCTGAAAGCGGATTCGCATAAGGGTCACCGCTCCCCTTATAAACCCACA
GGGTGGTCCCGGCACCTTTCACCGGCATTGTAGGATTTGGTACAGGCATAG
CGTCCTCACATTTCATAGGTAATGACATAAGTCAGATCGGCTGAACTCCACA
GGCCCGCATCATCGTCGCGCCGGTAGTCATAGCCGCTGGCCACCATACTGG
TGATCAAATCTGACAGTGCCGGGATATCGCTCATCACCGGATAAATCCGGG
ACTCCATCCACGCATCCAGCTCTGAATCCGGCACCTGAGCAGGCAGGAAA
ACTTCGATATGCAGCTCCGCCTGCCAGGTATCGCTGTCCAGCTCTTCGCCCG
TGTATTCAGCGCCGGTGAGATAAACGGCAACTGCCGGAAAATCCGCCTCAT
CAAAAACAGCGGGGCGACCATCAAAAAACGTCGCCCCGGTGTCATGCTTC
TCCAGTGCATCCAGTACGGCTGCACGGAGTTCAGTATGTTTCATCGCTTTAT
TACCATCCTCAGTTGATGCTGCAGCGCATAGCCCAACTCTTTCGGAAGACG
TTCACGCCGTATCCGCTCAATATTTTGTTTAAACGCCGTGGTCAGCGGCACC
GCCATCGGGATTTTCACCACATCAATGGGGTAACGGTTTTTCCCGGCCACA
CGCTGCATGACATGCCACCGGCCATTTTTCAGTTGCTGAATAAACGCGCCG
GGAATACGACGGTTTCCCACCACAAGCACGCTGCCGCCACCTTTCAGGGCT
GAACGCTGCCCCTTTTTACGACGCCTGCGTCGGGACAGGACAATCCGCGC
GTTACCCAGCTTTATTACGGGCAAATCCCCCCGGTTAACCTTGATTCTGGCC
TGCGGATTTTTGACCGTGGCCCTTTTCAGCCTGGCCCTTTCCTTTACCAGTT
TCCGGCGTACCTTTGTCTCACGGGCAACCTGTGACGCCGACTGCGATATCG
CGGATGAAGCAACGCGGTTAATGGCCATTGCGGCGGCACCGGGCACCGCC
GTTCTGCTGATACGGCTGAGGTTTTCAACGGCCTGCTCAAGACCTTTTATG
GCCATACATCCCCCTTTCAGCGGCGACGGTTAACGGCAGGCGGTACGCCCC

GCCCAAGCCAGAGATGACAGCTTCCGCCATCATCCGGCGAAATCCGGTCCA
CCCAGAAGTTTTCCTCGCCGATGGTCAGTGTGTCTCCACGCCGCAGCTGCC
GCACATCATCAGTCCGGACAAACAGGGACGGGCTGGAGCCTTCAACGCGC
ACCCCCTGTCCGGCATAGCTGATATTTTCAGGGTCATCAAAAACACCACGTA
TCACAGCACCGGACAGCTCACCGGATGTCATGGTGGCTGACGTTCCCATGT
ACCCGCGTATCGTTTCATCGGCGCGGGCAATGGCAGCATCGAACAGGTTAT
CGGAATCAGCCACAGCGCCTCCCGTTATTGCATTCTGGCCAGGCCATGTTCT
GTCATTTCGGCTGCCACACCGGCAGAGACACGAAACGCCGTTCCCGGCAG
CACAAATGCCACAGGCTCATCCCGCGTGGCGTGAAGTGCATCAGTATGCAG
CGTCACCAGTGCCACGACCGTGACCAGAGCAGCCGTATCAATCACGGTATC
CGGCTGCGCTGATACCACCTCATTTTCATGCCCGGTCAGCGCATTTTCCGGG
CTGACAGATGTGTCCTGACCGGCAGCGTCATCCGTGTCATCAAGCTCCTCT
TCCAGCTCTGCCACACGGAGTGCCAGTTCTTCTTTCGTCCCTGTCAGGCTG
ACATCACGGTTCAGTTGCTCACCCAGCACCTGAAGACGGGCAATCAGTTCA
TCTTTCGTCATGGACTCCTCCACAGAGAGAAAATGGCCCCGAAGGGCCATG
ATTACGCCAGTTGTACGGACACGAACGCATCAGGGTCAGCCAGCAGCATC
AGCGGTGCTGACTGAATCATGGTGAACTCACGCGCCGGATCGCCGGTGGT
CACCCAGTTTTTCGGGTAGCGGGCAGAGGCGTTAATACCTTCGCGCTGTGC
GTCCGCATCCTGAATGCAGCCATAGGTGCGCAGACCGCGTGCCTGAGTGTT
CCCCAGCACCATCGTGTTGTCCGGAAGGAAGTTCTTTTTGACGTCGTTTTC
CACATACTGTCCGGAATACACGATGATCGCCGTATCGCCATACATCCCCTTAT
AGGACACCGCTTCGCCCAGGTCTTTTACCGCTGTCTCCAGCTCGGAATTAG
AGCCGCGACGGGTATCCAGCTTCTCCTTGACGGCTTTGAAGGAACGGAAC
AGCGCCCAGCCTTTCGGATCAAACACGATGATATTCACCACACCGCTGGCG
TTCAGCGCGTAGGCTTCGATATCGTCGGTCGGGTCATACGTGGACTTGTCAC
GCTTGCTCCACTCCGTACCGCCGGACTGCGTGATGTTGTTCGCCACACTGC
GGCCCATATCCACTTCAACCGGATCGAAGGCTTCACCGGTCATGGTGTATTT
GCCCTTAAGCACGGCAGAAACTGCCTGCATCTCTTCGACCTGAGCAATGGC
CAGCTCTTCGTCTCGCATGTTCTGCAGGATGATGCGACGGCGGCGGTAAGC
CGGGTCCGCCAGATTCTGTGGATCTTCATCCGGCAGGCGACGCAGGGTCAT
CTGCGGATTCACCTCATGCTTGGGTTTGACATATCCCGGTGTAAATTCAGAG
GTGGAGCCGCCACGGGAGCGGATAACCTCACCGGAAACAATCGGCGAAAC
GTACAGCGCCATGTTTACCAGTCCCGGAATTTGTGAGAGATAGACTTTCTCC
GTGGTGAAGGGATAGCTCTCACGGAAAAAGAGACGCAGAAACAGCGGAT
CAAACTTAAATTTCTTCTCATTTGCCGCCAGCAGCTGGGCGGTTGTGTACAT
CGACATAAAAAAATCCCGTAAAAAAAGCCGCACAGGCGGCCTTTAGTGAT
GAAGGGTAAGGTTAAACGATGCTGATTGCCGTTCCGGCAAACGCGGTCCG
TTTTTTCGTCTCGTCGCTGGCAGCCTCCGGCCAGAGCACATCCTCATAACG
GAACGTGCCGGACTTGTAGTACGTCAGCGTGGTGCTGGTCTGGTCAGCATC
AACCGCCAGAATGCCAACGGCAGCACCGTCGGTGGTGCCATCCCACGCAA
CCAGCTTACGGGTGGAGGTATCCAGCATCAGCGGGGTCATTGCAGGCGCTT
TCGCACTCAATCCGCCGGGCGCGGTTGCCGTATGTGCCGGGTCACTGTTGC
CCAGCGGCTGGTAATGGGTAAAGGTTTCTTTGCTCGTCATAAACATCCCTTA
CACTGGTGTGTTCAGCAAATCGTTAACGGCATCAGATGCCGGGTTACCTGC

AGCCAGCGGTGCCGGTGCCCCCTGCATCAGACGATCCAGCGCAGTGTCAC
TGCGCGCCTGTGCACTCTGTGGTGCAGCTGCCAGAATGCGGCGGGCCGTTT
CCACGGTCATACCGGGGGTTTCGGCCAGCACGCGTGCCTGTTCTTCACGTC
CGTGAGCCTCCTCACAGTTGAGGATCCCCATAATGCGACTGTTTTCTGCCG
CAACCGCTGCGGTGATCTGCGCGTTTACGTCCGGCTGCGCCGCGCTGGCGT
TCTCGCCCTCCGTCGCTTGCACCACGCCAGTAACGTCAGCCTGCGAAGCAG
TGGCTGAAACAGTTGTTGATTGAGTCTCTTTGGTCATTCGCCCTCCTGAGA
GACGGGATTTACGTGCATCCAGTGCATCACGCATGACGGTGATCGCATCGG
TACTGTTAACAAGTTCATCAGCCAGTCCGGCATCAATGGCCTCCTGACCGC
TGTACACTGCAGCCTCGGTATCCAGCACAGCCTGCACAGACAGGCCGGTAT
ATGCCGACACCTTCTGTGCAAACATCCGGCGGGTTGCATCCATCCGGGACT
GCAGTGTTTCCCGGACATCATCCGGTAGATGGCTGTAGGGGTTGCCATCCA
CCTTATGGCTGCCGCTGTAAATCAGCGTGATTTCCACGCCCTGTTTCTCCAG
CGCAGCGCCGTAATTACTGTGAGCCATCATGACGCCGATGGAGCCTGTCCG
GGCGGTCTGCGTGACCAGACGCCGGGAGGCGGCACTGGCAAGCAGCTGA
CCTGCGCTGCAGTTCATGTCATTGGCCAGCGCCCATACCGGCTTTATGTCAC
GCACACGGGCGATGATGTCAGCGCAGTCAAATGCCCCCGCCACCATTCCGC
CTGGCGTATCCATATCGAGCAGAATGCCGTCCACCATCGGGTCACTGGCAG
CCTGTTGCAGACGGGCGATAATGCCGTTGTAACCGGTCATTCCCGAATACG
GCTGCAGCGCCCGCGTCCGACTGACCAGCGTACCGGACACCGGCAGCACG
GCGATGCCGTTCATGACCTGATAACTGCGGGCCTGTCGTGGTCCGTCATCAT
CACCGGATAACGCCAGCGCCGCGGGTGCCTCTCCGGCAGTCAGGCTGTCG
CCGGATACTGCATCCGTCAGGCGGCTGATCCCAAGCTGGCCTGCAAGCGCA
CAAAAGAAAACCCGCGCATAGGCGGGTTCAAGCATCAGCGGCTCATTAAA
AGCCATGCTGGCAATATGCGGGAGATTACGCAGCTCTGCTGTCACTCTTCTC
CTCCTCTGTTGATTGTCGCAGCCCGGATTCAAATGCCGCAGCCGCCCAGGC
GGGCGGTTTAAGACCGGCTGCACGGCGCTCCATCGTTTCACGGACCTGCTG
GGCAAAAATTTCCTGATAGTCGTCACCGCGTTTTGCGCACTCTTTCTCGTAG
GTACTCAGTCCGGCTTCTATCAGCATCACCGCTTCCTGTACTTCTTTCAGAC
CATCGATGGCCATACGACCGGAGCCTATCCAGTCACAGTTCCCCCAGGCGC
TGCGGGCTTCCTGAAAGCTGAAACGCGCTTTTGAAGGTAACGTCACCACG
CGGCGAACGATGGCCTCTTCCAGCCAGCACAGAAACATCTGGCTCGCCTG
ACGGGATGCGACGAATTTTCGCCGCCCCATAAAGTACGCCCACGACTCGTT
CGCACTGGCCCGTGCCGTGGAGTAGCTCATCTGGGCGTAATTCCGAGAAAG
CTGCTCATACGAGACACCCAGCCCGGCAGCGATATACCGCAACAGTGACTG
CTCAAACACGGAGTAGCCGTTATCCGTATCCTGAGCCGTCTGCAGGTTCAG
TGAGTCACCCGGCATCAGGTGCGGCACTTTTGCGCCTCCCAGCCGGACCG
GTGCTGCGGCGTAATACGCGGCAATTTCACCAATCCAGCCGGTCAGCTTGT
CCCGCTGCTCCTGACTGTTCGCGCCCAGAATAAAATCCATCGCTGACTGCG
TATCCAGCTCACTCTCAATGGTGGCGGCATACATCGCCTTCACAATGGCGCT
CTGCAGCTGCGTGTTCTGCAGCGTGTCGAGCATCTTCATCTGCTCCATCAC
GCTGTAAAACACATTTGCACCGCGAGTCTGCCCGTCCTCCACGGGTTCAAA
AACGTGAATGAACGAGGCGCGCCCGCCGGGTAACTCACGGGGTATCCATG
TCCATTTCTGCGGCATCCAGCCAGGATACCCGTCCTCGCTGACGTAATATCC

CAGCGCCGCACCGCTGTCATTAATCTGCACACCGGCACGGCAGTTCCGGCT
GTCTCCGGTATTGTTCGGGTTGCTGATGCGCTTCGGGCTGACCATCCGGAA
CTGTGTCCGGAAAAGCCGCGACGAACTGGTATCCCAGGTGGCCTGAACGA
ACAGCTCACCGTTAAAGGCGTGCATTGCCACACCTTCCCGAATCATCATGG
TAAACGTGCGTTTTCGCTCAACGTCAATGCAGCAGCAGTCATCCTCGGCAA
ACTCTTTCCATGCCGTTTCAACCTCGCGGGAAAAGGCACGGGCTTCTTCCT
CCCCGATGCCCAGATAGCGCCAGCTTGGGCGATGACTGAGTCGGAAAAAA
GACCCGACGATATGATCCTGATGCAGCTGGATGGCGTTGGCGGCATAGCCG
TTATTGCGTACCAGATCGTCTGCGCGGGCATTGCCACGGGTAAAGTTGGGC
AGCAGGGCTGCATCCACACTTTCACTCGGTGGGTTCCACGCCCGCAACTGC
CCACCAAATCCGCTGCCACCGCCGTGATAACCGGCATATTCACGCAGCGAT
GTCATGCCGTCCGGCCCCAGAAGGGTGGGAATGGTGGACGTTTTCATACAT
AAAATCCTGCAGGTCCCCTGCGTCGCTGTGTCATGCCGGTCTGCACTTCCA
GCTCCGCAATGTATTTTTTCAGGTCAGACACGGAAGTGGCCGTAAACTCCA
CTCTCCGTCCGTCTTTCTGTACCGTTGCCACCCGTTTTCCTGTCATCAGGTC
ATGCAGTGCCGCACGGGCAGCGGCAAGTTCTTCCTGTCGCGTCATTCATCC
TCTCCGGATAAGGCACGGGCGTAATCTGCCAGTGTTTTCTTGTTGGTTGCTG
CACCATCCTCTTCCTGCAGGCTCGCCAGCAGTGCACTGAGATCCAGCTGCC
AGCGGGAAATACTGATGCGCAGCGCCGCCAGCGCATAGACGAAGCAGTCG
AGCGCCTCATTGCGTCGCTTTTTGCTGTCCCACAGTATTTTTTTCCTGCCATC
CACCCATTTTTCGACCTGCTCTTCAGCAGTCAGCTGCTGCGCTTCGGTCAG
ATCAAAAATATCCGGGTTATTCGGGAAGTGAACGGCACCGGGAAGCGGTTC
ATCCCCTTCCGGCGTCAGTGTGAAGCGGTTATAAATCTGCTCTTTCGCGGTA
TCCGTACCGATTTCGGTAAGGTAAACCCCGTTTTTGTTTCGCTTACGTGGCA
TGCTGGCCACCGGCTTTCCGTAGACGGATGCCCCTTTAATGGGGATCACCC
GGAACAGCCCATGCTTTTTCGAGCGTTCATACACAATGGTCGGGTCAATCC
CGCCAGTATCCCAGCAGATACGGGATACCGACATTTCTGCACCATTCCGGC
GGGTATAGGTTTTATTGATGGCCTCATCCACACGCAGCAGCGTCTGTTCATC
GTCGTGGCGGCCCATAATAATCTGCCGGTCAATCAGCCAGCTTTCCTCACCC
GGCCCCCATCCCCATACGCGCATTTCGTAGCGGTCCAGCTGGGAGTCGATA
CCGGCGGTCAGGTAAGCCACACGGTCAGGAACGGGCGCTGAATAATGCTC
TTTCCGCTCCACCATCACCTCAGCATCCGGACGTTCGCCGATTTTCGCTTCC
CATGTCTCACCGAGCGTGGTGTTCACGAAGGTTTTACGTTTTCCCGTATCCC
CTTTCGTCTTCATCCAGTCTTTGACAATCTGCACCCAGGTGGTGAACGGGC
TGTACGCCGTCCAGATGTGAAAGGTCACACTGTACGGCGGCTCAATCTCTT
CACCGGATGACGAAAACCAGAGAATGCCATCACGGGTCCAGATCCCGGTC
TTTTCGCAGATATAACGGGCATCAGTAAAGTCCAGCTCCTGCTGGCGGATG
ACGCAGGCATTATGCTCGCAGAGATAAAACACGCTGGAGGGGTCATCCGG
CGTCCATTTGAGGCCAAACGGCGTCTCTTTGTCGCCAAATTTAAGATACTG
CTCCTCCCCGCAGTGCGGGCAGGCAACATGAAAACGCATAAAATGCGGGG
ATTCACTGGCTGCACGCTCAATCTGGCAGGTGCCTCTCACTTTGGGCGTGG
AGCCACGGATGGACTTTGGCCAGACCGAGCCTTCAATACGTTTGTCGCCAA
GGAACGTCGGAGAGCCTTCCTGTTCAATATCCTCATCAAAGGCAGCAAGTT
CATCATAACCCGCCACATCCACCGACTTTTCACGGTAGTTTTTTGCCGCTTT

ACCGCCCAGGCACCAGAAGCCACGACCATTGGAAAAACGCTTCATAGTGA
GCGTGTTATCCCGGTGCTTTTTGCCATACCACGGAGCCAGCGCCAGCAGCG
ACGGAATATCGCGGATGGTCGGCTCAACGTGGGTTTTCATAAAGTTCTCGG
CATCACCATCCGTCGGCAACCAGATAAGGGTGTTGCGCTGCCTATGCTCTAT
AAAGTAGGCATAAACACCCAGCAGCATTTTGGAATAACCGACACGGGCAG
ACTTCACCACATTCACCTCACGGATGTAGTCGCTGCCCATCGCATTCATGAT
GGCCCGCTGAAAGGGCAGTGTTTCCCAGCGCCCTTCCTGGTATGCGGATTC
TTTCGGGAGATAGTAATTAGCATCCGCCCATTCAACGGCGGTCTGTGGCTCC
GGCCTGAACAGTGAGCGAAGCCCGGCGCGGACAAAATGCCGCAGCCTGTT
AACCTGACTGTTCGATATATTCACTCAGCAACCCCGGTATCAGTTCATCCAG
CGCGGCTGCTTTGTTCATGGCTTTGATGATATCCCGTTTCAGGAAATCAACA
TGTCGGTTTTCCAGTTCCGGAAAACGCCGCTGCACCGACAGGGGGATCCC
GTCGAGAATACTGGCAATTTCACCTGCGATCCGCGACAGCACGAAAGTACA
GAATGCGGTTTCCACCACTTCAGCGGAGTCTCTGGCATTCTTCAGTTCCTG
TGCGTCGGCCTGCGCACGCGTAAGTCGATGGCGTTCGTACTCAATAGTCCC
TGGCTGGAGATCTGTCTCGCTGGCCTGCCGCAGTTCTTCAACTTCCCGGCG
CAGCTTTTCGTTCTCAATTTCAGCATCCCTTTCGGCATACCATTTTATAACGG
CGGCAGAGTCATAAAGCACCTCATTACCCTTGCCACCGCCTCGCAGAACGG
GCATTCCCTGTTCCTGCCAGTTCTGAATGGTACGGATACTCGCACCGAAAAT
GTCAGCCAGCTGCTTTTTGTTGACTTCCATTGTTCATTCCACGGACAAAAA
CAGAGAAAGGAAACGACAAAGGCCAAAAAGCCCGTTTTCAGCACCTGTC
GTTTCCTTTCTTTTCAGGGGGTATTTTAAATAAAAACATTAAGTTACGACGA
AGAAGAACGGAAACACCTTAAACCGGAAAATTTTCATAAATAGCGAAAAC
CCGCGAGTCGCCGCCCCGTAACCTGTCGGATCGCCGGAAAGGACCCGCAA
AATGATAATAATTATCATCTACATGTCACAACGTGCATCTACGCCATCAAACC
ACGTCAAATAATCAATTATGACGCAGGTATCGTATTAATTGATCTGCATCAAC
TTAACGTAAAAACAACTTCAGACAATACAAATCAGCGACACTGAATACGGG
GCAACCTCATGTCAACTAAGAACAGAACCCGCAGAACAACAACCCGCAAC
ATCCGCTTTCCTAACCAAATGATTGAACAAATTAACATCGCTCTTGAGCAAA
AAGGGTCTGGGAATTTCTCAGCCTGGGTCATTGAAGCCTGCCGTCGGAGA
CTAACGTCAGAAAAGAGAGCATATACATCAATCCAAAGTGATGATGAATAA
ACATCCCGGTTTCTTCCACCATCGCACCGGAAAAGCGACTATGAGGGTAAC
CCTGCGTCTGTCAGCACAGTAAAACCCGGTGTGCATCGTTTTTGATTATTCC
CGCACACTCACGCAGAAGGAATTCCCCGTCGGGCTACGGTCATGGTTAATG
CGGGAATACGGCGACGATACAGCGCAGCTAAAAGGGTAATGGACAGAAAG
AGCGGTTTATTTCATTCCACAGGATTCTGAGTGCCCCCCTCCTCCAATAGGC
TGAGCATCCACCTATATAGTTTTAATTTTCATCAATCCATTTAACTATCGTTTA
ATTGTTGTCACATAGGATTCTGCCGTTTTTAACAATGCAGGATAATAAGATG
AAAAAAATGTTGTTTTCTGCCGCTCTGGCAATGCTTATTACAGGATGTGCTC
AACAGACGTTTACTGTTGGAAACAAACCGACAGCAGTAACACCAAAGGAA
ACCATCACCCATCACTTCTTCGTTTCGGGAATTGGACAGGAGAAAACTGTT
GATGCAGCCAAAATTTGTGGCGGCGCAGAAAATGTTGTTAAAACAGAAAC
CCAGCAAACATTCGTAAATGGATTGCTCGGTTTTATTACTTTAGGCATTTATA
CTCCGCTGGAAGCGCGTGTGTATTGCTCACAATAATTGCATGAGTTGCCCAT

CGATATGGGCAGCTCTATCTGCACTGCTCATTAATATACTTCTGGGCTCCTTC
CAGTTGTTTTTGCATAGTGATCAGCCTCTCTCTGAGGGTGAAATAATCCCGT
TCAGCGGTGTCTGCCAGTCGGGGGGAGGCTGCATTATCCACGCCGGAGGC
GGTGGTGGCTTCACGCACTGACTGACAGACTGCTTTGATGTGCAACCGAC
GACGACCAGCGGCAACATCATCACGCAGAGCATCATTTTCAGCTTTCGCAT
CAGCTAACTCCTTCGTGTATTTTGCATCGAGCGCAGCAACATCACGCTGAC
GCATCTGCATGTCAGTAATTGCCGCGTTCGCCAGCTTCAGTTCTCTGGCATT
TTTGTCGCGCTGGGCTTTGTAGGTAATGGCGTTATCACGGTAATGATTAACA
GCCCATGACAGGCCGACGATGATGCAGATAACCAGAGCGGAGATAATCGC
GGTTACTCTGTTCATTGCTGACCCCACAAACAGATTTCACGCTCAATCTCAC
GACGAGTCATCAGGCCTTTCCATTGCTTACCGCCAGCATATGTCCAGCGAC
GTAGCTGATCACATGCGCCTTTGATATCGCCCTGGTTTATTTTGCGAAGAAG
CGTCGATGTTCTGAAATTGCCAGCACCCACGTTGTAAACGAATGAGTAAAG
AGCGCCGCGCATTGTTTCCGGTATATCGACTTTGATGTACGGGTTAATTTGT
CTGGCGACCGTGGCAAGGTCTTTATTCAGGAGGGCTTTGCATTCTGCTTTG
GTATACGTTTTACCGAGCATGATGTCTTTTCCTGTATGCCCGTGACATACAGT
CCATACACCAACAATATCTTTGTATGGTATGTAGCTGACACCTTCCAGACCA
TCGTTACCACTTGGGCCAGTAATTAACACTGATGCTATAGCAATTGCTCCGC
CACCAATAGCAGCAGCAACGGCTTTTCGTAATGATGGAGGCATTATTCACCT
CTCGCAGCCTTGCGCTTATCTTCTTTAATCTTGAAATAAAGGTTTGTCAGGT
ACGTCAGCAGGCCAAATACCAGGCTACCCAGCACACCTATTGCTGCCCACT
GTGAGGGCGTGACTTTATCGAGCAGCTGTAAAAACCAGTAACCGGCACTA
CCTGCTGAGGTGCCATAGGCGACACCCGTTGTTAACTTATCCATGGATTTCA
TAACCCCACCTCGCAGACAAAGCGGGTGTAAATTGAGGGAATACAACGTAT
CGCAAAAAAGCAGAAACGTAACAGACTCGGAGTCAGTGAATAACTCAGGT
ATTGAGTTATCAGCTAATATCGAGACTCAAAAAATGGAAAAACCAGCTCGA
CGGCGGGTTTAAGCTGTGTGACGAAGTAACCACTCTTAACAGCATAACCAA
TTTTTTACGTACGTAAACCACTGAATGATATTTATGAGAATGCTACCGAGTG
TTCAAAACACCACCACAAATACATAAGAAAACCTCAACAAATAACCAATAA
ATAATTTCAGACGTTATTTTTAGTTGATTTAAATTAAACTGCCGAATTATAGA
ACCTCCATAAATAACAACCATTAATATAAATTAGCTAATAGGTTTATTTTTGT
TCAAATAAGAGCCATAAATAGGTTTCGATAGAAAAAGTTCAGATAAAAATA
GAGATCTACTTCACAAATTAAATGAGAAACTAAAACTTACATCTTGAAATAA
TCACATTGATTAGATGAATATTTATCGCGCAGTGACATCATTTTTTAATAATA
GTTCAAAAAAAAGGGCTCACGATGAAAAAATTAACAGTGGCAATTTCTGC
TGTAGCTGCATCAGTACTGATGGCGATGTCTGCTCAGGCAGCTGAAATTTAT
AATAAAGACAGTAACAAGCTGGATCTGTACGGGAAAGTTAATGCCAAGCA
CTACTTCTCCTCTAATGATGCAGATGATGGTGATACTACTTATGCCCGTCTTG
GCTTCAAAGGTGAAACCCAAATCAACGATCAACTGACTGGTTTCGGTCAG
TGGGAATATGAATTCAAAGGCAACCGCGCTGAATCTCAAGGTTCCTCCAAA
GACAAAACCCGTCTTGCATTTGCAGGCCTGAAATTCGGTGACTACGGCTCA
ATCGATTACGGCCGTAACTACGGTGTAGCATACGACATCGGTGCGTGGACT
GACGTTCTGCCAGAATTCGGTGGCGATACCTGGACCCAAACAGATGTGTTC
ATGACTGGTCGCACCACTGGTGTTGCAACCTATCGTAACAACGACTTCTTT

GGTCTGGTTGATGGTCTGAACTTTGCTGCTCAGTACCAAGGCAAAAACGAT
CGTAGCGATTTCGATAACTACACTGAAGGTAACGGTGATGGCTTCGGTTTCT
CTGCTACCTATGAATACGAAGGATTCGGTATCGGTGCAACTTATGCGAAATC
TGATCGTACCGACACTCAAGTTAATGCAGGGAAAGTTCTTCCTGAAGTATT
TGCTTCCGGTAAAAATGCAGAAGTTTGGGCCGCAGGTCTGAAATATGACGC
TAACAACATTTACCTGGCCACTACCTATTCTGAAACCCAGAATATGACTGTA
TTTGCTGATCACTTCGTTGCTAATAAAGCCCAAAACTTCGAAGCTGTTGCA
CAATATCAGTTCGATTTCGGTCTGCGTCCGTCCGTTGCTTACCTGCAATCTA
AAGGTAAAGATCTTGGAGTATGGGGCGATCAGGACTTAGTCAAATATGTTG
ATGTAGGTGCAACCTATTACTTCAACAAAAATATGTCTACTTTCGTTGATTAC
AAAATCAACCTGCTTGACAAAAATGACTTCACTAAAGCACTCGGTGTAAGC
ACTGATGACATCGTTGCTGTAGGTCTGGTTTACCAGTTCTAATCTGATTACG
AAAAAGATATGTTGCGGGAGGCGTTGCCTCCCCAACATATAAGTGGCTCCC
TCAAGCCACTTCCTTTAGAAGCACAACCTTGCTTCTAACTATATAAACCTTC
TGTTATATATTACCCTTTATTTTTGGGGGCGTTTCAACGCCCCATTTTTAATAA
TTTTTAGTAAACAATTGGCATATTAATTAGAGTTATTAACAACGATATCCATC
TCTAACCGGATATCTAATGCCATTAACATCCCTTCAATTATGCCCTCAGCCTT
CTGTAACCTTTTCCCGATATAACCATCAGAGCAGCAATGCTTACCTGCCAGT
GACATGAATGTCATACCGACTACATAATAATCTACTAATAAATCGTGCAAATC
GCTGTTGTTCTTTTTCAGACGGGCCATGCACCCGCAAATAATCATCGCGTCA
TCGTCACAACATTGCGGGCGAGATTTTACTTTTGAAGTAATTAATCCCTTAA
AACCGGCGGCAATGGACGACCAGGTCACATCTTCATGATTATTAGCCGCCC
ACGCTCCCCAACGCTCAAGAACCATCTGAATATCACGCATCAACTTACTCC
ACAAAAATCAGACCAGAACGCCAATTACAAGCAAAAATCAACGAAACAGT
ATTAGTTGATTGTTATCTCTGACTTCATACTCCTGCTCCTGTCAGGGTTTTGG
CGTAATTCTTCAGTATTCGGTAATCGGTCAAAACAGAACCGGGGAAACGAT
ATAAGCGCAGACGCCCCCAGCGGTGGCGAAGACGTTCTGCCATATAAAACT
CAAACATCATTCATTCCCCATTTCGGTGATTTTTATTCCAAGCCGTCCGCCTG
GTACTTTCACACCACGAATTACGCGAATGTCATCGAATTGCTCGTCGTCTTC
CGCAAATCCGGCGTGGATAAGGGAGTCGAGTAAACCTTTCAGGATGTTATC
GAGGTCGCGGCGGCGGGAGTCTGGAACGTCTGCGATGACTTTGATGCGGA
GTCGTGATTTGGTGAAAATGTCTAACTTGAGTTGGCGGATGATTTGCTGTAC
ATCTTTTCGGTATTTCTGGCCTTTATCGCTGATGTAGTATTGGCTTCCCCGTC
TTCGCCAGTAGGTGTTCAGCGACGGTGGGTATGGAAGCACAAACTGATATT
CGTTCATTGGACAAACACCCTCCCATTGTTAACCAATTTTTTAAGGGTAAGC
ACAATGGCCCGATCCATTTCAGCCCTTCGCTCTTCCCTGTTCATATCTCTCCC
ATTATCAATTCTTGAATGACAATCAACACATAGCGCAGCAGTAAGGCAATCA
TCTACTTTAATCCCAACCCCCTTCCCTTCATTTCTGTGAGCGGCCTGAACTC
CGTATCTTCCGCAAAGAACGCAAAATTCAATATCCCTGACTGCCTGAAGCC
ATTTACTGCTCCGGTATATTGCCATCTGAGATATCTCCGTTCGGATCACGATA
AATCAGCCATTCATCCACGCATTCAGAACATGCGTAGGTTTCCTCTGGCGTT
AGTTCCTTCGTGCAGCCGGCGCACAGCGTTCGACGTATGCTCTGCTGCTCG
TACTCTTGAGGCCATGGGG
